# Expansion of epileptogenic networks via neuroplasticity in neural mass models

**DOI:** 10.1371/journal.pcbi.1012666

**Published:** 2024-12-03

**Authors:** Elif Köksal-Ersöz, Pascal Benquet, Fabrice Wendling

**Affiliations:** Univ Rennes, INSERM, LTSI UMR 1099, Rennes, France; Centre National de la Recherche Scientifique, FRANCE

## Abstract

Neuroplasticity refers to functional and structural changes in brain regions in response to healthy and pathological activity. Activity dependent plasticity induced by epileptic activity can involve healthy brain regions into the epileptogenic network by perturbing their excitation/inhibition balance. In this article, we present a new neural mass model, which accounts for neuroplasticity, for investigating the possible mechanisms underlying the epileptogenic network expansion. Our multiple-timescale model is inspired by physiological calcium-mediated synaptic plasticity and pathological extrasynaptic N-methyl-D-aspartate (NMDA) dependent plasticity dynamics. The model highlights that synaptic plasticity at excitatory connections and structural changes in the inhibitory system can transform a healthy region into a secondary epileptic focus under recurrent seizures and interictal activity occurring in the primary focus. Our results suggest that the latent period of this transformation can provide a window of opportunity to prevent the expansion of epileptogenic networks, formation of an epileptic focus, or other comorbidities associated with epileptic activity.

## Author summary

Repetitive epileptic activity leads to a large variety of plastic changes in brain. Understanding the mechanism and its time course can indicate windows of opportunity for preventing the devastating impact of focal epileptic activity on brain networks. Animal models of focal epilepsy have underlined the role of NMDA receptors and deregulations in the GABAergic system in the expansion of epileptic activity. Here we develop a plastic neural mass model integrating physiological and pathologic plasticity. Our results show that neuroplasticity mediated by epileptic activity increases synaptic efficacy between epileptic and healthy regions, and causes irreversible changes in the GABAergic system in the healthy region. The model gives mechanistic insights into the physio-pathological impact of focal epileptic activity on large-scale networks and suggests that seizures may beget seizures.

## Introduction

The most general definition of neuroplasticity is the ability of neural networks to reorganize over time, which can take place under physiological and pathological conditions. Activity dependent plasticity is the ability of synapses to strengthen or weaken, in response to increases or decreases in their activity. Under physiological conditions, it is accompanied by many regulatory mechanisms through which the excitation/inhibition balance is preserved (for examples [1–6]). However, epileptiform discharges drive not only physiologic synaptic plasticity but also pathological plasticity, which disturbs the excitation/inhibition balance, and can cause expansion of epileptogenic networks during epileptogenesis [7–9]. The aim of this article is to investigate the expansion of epileptogenic networks with a mathematical model of physio-pathological plasticity under recurrent epileptic activity.

Synaptic plasticity occurs on multiple timescales at both presynaptic and postsynaptic sites [10]. Short-term plasticity (STP, in milliseconds) refers to transient changes in the probability of neurotransmitter release at the presynaptic site [11]. Long-term synaptic plasticity (in hours or days), although generally considered as postsynaptic alterations, is related to both (i) the insertion/removal of alpha-amino-3-hydroxy-5-methyl-4-isoxazolepropionic acid (AMPA) receptors (AMPARs) at the postsynaptic site and (ii) the change in the probability of neurotransmitter release at the presynaptic site [12].

Postsynaptic long-term potentiation (LTP) and long-term depression (LTD) depend on the calcium concentration at the postsynaptic site [13]. In excitatory (glutamatergic) synapses, an increase in postsynaptic calcium concentration through activation of synaptic N-methyl-D-aspartate (NMDA) receptors (NMDARs), metabotropic glutamate receptors, voltage-gated calcium channels, etc. is necessary and sufficient for synaptic plasticity (see [12,14] and references therein). Calcium-dependent activation of secondary messengers, such as nitric oxide, endocannabinoids, etc., triggers presynaptic long-term plasticity [15,16]. The late phase of long-term plasticity, referred to as maintenance or consolidation, occurs via new protein synthesis and gene transcription [12]. We refer to these processes of postsynaptic long-term plasticity, presynaptic long-term plasticity and consolidation as *physiological plasticity*.

Epileptic discharges that cause glutamatergic spillover have a twofold impact. First, epileptiform activity strengthens excitatory synapses by increasing the number of AMPARs [17,18], as in the case of LTP. Second, a high amount of glutamate released during epileptiform activity can activate extrasynaptic NMDARs that cause pathological changes in GABAergic signaling, through inactivation of functional potassium chloride co-transporters 2 (KCC2) and internalization of GABAergic receptors [19,20]. KCC2 controls the outflow of the chloride ions from the intracellular space to the extracellular space and maintains the GABAergic reversal potential. Calcium ions flowing through extrasynaptic NMDARs bind on calpain that inactivates KCC2 [20–22]. Reducing the number of functional KCC2 results in the increase of the intracellular chloride concentration and changes GABAergic responses from inhibitory to excitatory. KCC2 dysfunction is related to epileptic activity in the naive site [7], and it is one of the factors contributing to epileptic activity in the mature brain [23].

The extrasynaptic NMDAR activity also affects tonic inhibition that is mainly mediated by the *α*_5_ GABA_A_ receptors in the hippocampus. Overexpression of GluN2B NMDA receptors, which are located mostly in extrasynaptic regions, downregulates the expression of *α*_5_ GABA_A_ receptors expression, promotes their internalization, and therefore, reduces tonic GABAergic [24] (but see [25] for limbic epileptogenesis). We refer to these two mechanisms, i.e. reduced KCC2 and internalization of extrasynaptic GABAR, as *pathological plasticity*.

After several seizures physiological plasticity (LTP, LTS) seems to be occluded. However, pathological plasticity can increase the susceptibility of a brain region to epileptic discharges, expand epileptic network, and generate a secondary (mirror) focus. Secondary focus refers to epileptogenesis in a naive area induced by repetitively uncontrolled epileptic seizures from a primary seizure focus [26,27]. Independent epileptic activity in the contralateral area in humans suggests that mirror focus develops via a kindling process mediated by the primary focus. In animal models of epilepsy, for instance, under kindling [28] or kainite application [7] to one of the hippocampi (primary focus), secondary focus appears in the contralateral hippocampus due to recurrent epileptic activity in the primary focus. Khalilov’s elegant work in the immature brain [7] showed that activation of NMDARs and long-term alterations in GABAergic synapses are responsible for chronic epileptogenesis in the secondary focus. Despite the key role of plasticity in expansion of epileptogenic networks, there is a lack of computational modeling studies of neuroplasticity under epileptic activity.

Previous modeling studies of calcium-mediated postsynaptic LTP/LTD have mainly focused on cellular activity [29–34] (see [35] for a mean-field approximation), as well as presynaptic long-term plasticity [30,33,36,37]. Recently, Chindemi et al. [30] developed a detailed cellular model of the neocortex that integrates the calcium-mediated presynaptic and postsynaptic plasticity mechanisms. However, the literature related to modeling calcium-mediated plasticity with neuro-physiologically plausible neural mass model (NMMs) remains limited, and up to date up to date no computational model implements the activity dependent pathological plasticity. We identified two studies on this phenomenon in the context of magnetic stimulation [38,39]. The originality of this study is to differentiate the impact of physiological plasticity and the pathological plasticity induced by epileptic discharges on epileptogenicity of NMMs. Our computational model concentrates on the physio-pathological plasticity under epileptic activity in a network of two unidirectionally coupled NMMs [40], each representing an epileptic and a non-epileptic brain region, where the epileptic one perturbs the non-epileptic one. First, we study how synaptic connections evolve under epileptic activity. We then investigate the pathological plasticity triggered by the physiological plasticity. The results suggest that the interaction between physiological and pathological plasticity can explain the expansion of epileptogenic networks and the formation of secondary epileptic foci.

## Methods

### Mesoscopic model of physiological short- and long-term plasticity

**Fig 1:**
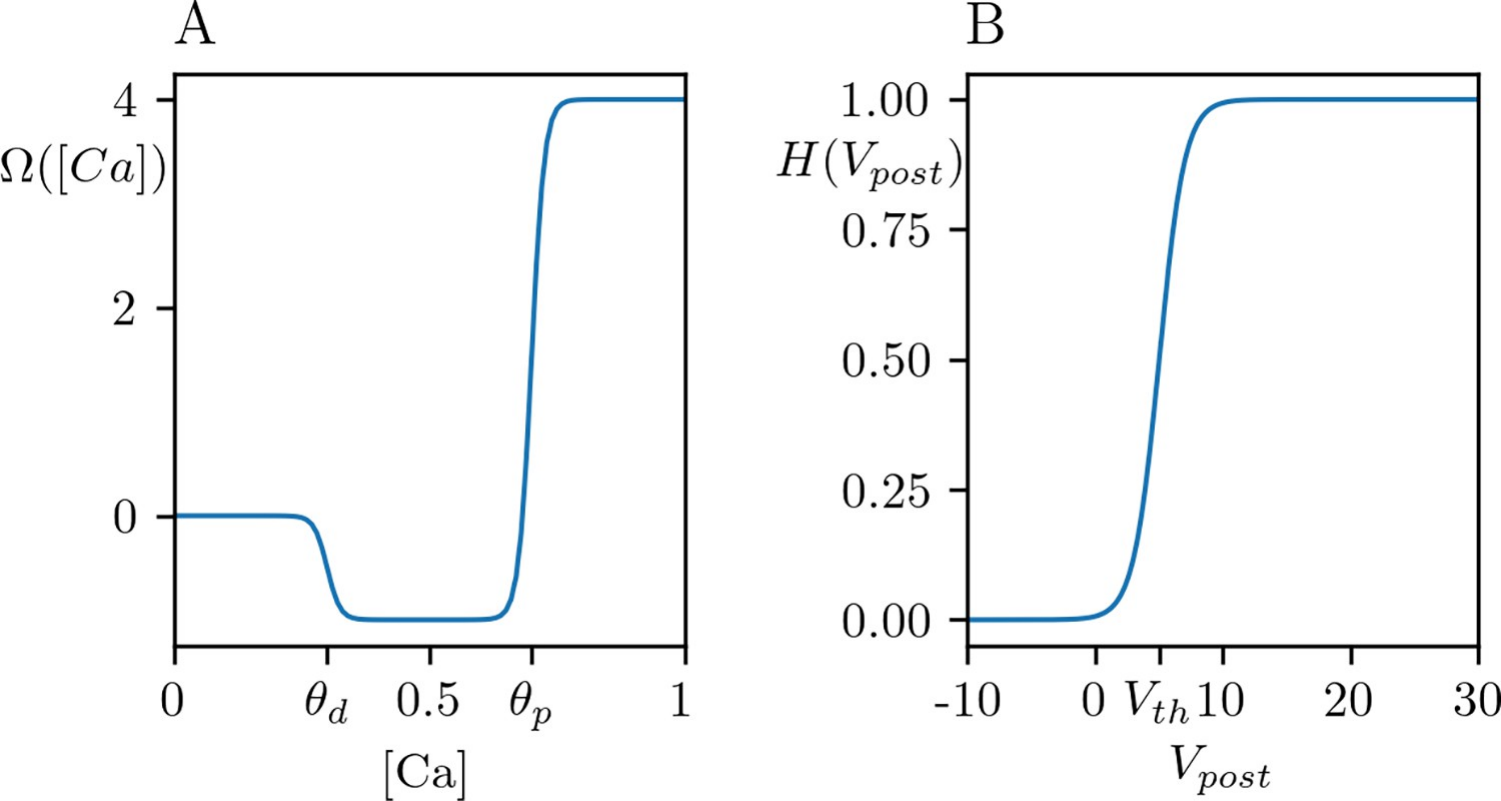
Synaptic efficacy and activation functions. (A) Calcium dependent synaptic efficacy function, $\Omega ([Ca])=-\Omega_{d}([Ca])+\Omega_{p}([Ca])$ (2), and (B) activation function, *H*(*V*_*post*_) (5) for the parameter set given in Table 1.

The seminal study by Shouval et al. [29] has proposed a calcium control hypothesis for postsynaptic plasticity, in which intra-cellular calcium concentration controls the rate of change of synaptic efficacy. The proposed model in [29] was then developed to account for the synaptic consolidation in [32] by introducing a variable *ρ* that describes the state of the synaptic efficacy. We combine both formulations as:

$$\frac{d\rho }{dt}=\frac{1}{\tau_{\rho }}(-\rho (1-\rho )(\rho_{*}-\rho )+(1-\rho )\Omega_{p}([Ca])-\rho \Omega_{d}([Ca])),$$ (1)

where *τ*_*ρ*_ is the time constant of the synaptic variations, ρ_*_ = 0.5 is the boundary between the depressed (*ρ* = 0) and potentiated (*ρ* = 1) states. The functions Ω_*p*,*d*_([*Ca*]) describe how the synaptic efficacy is controlled by the calcium concentration:

$$\Omega_{p,d}(\left[Ca\right])=\frac{\gamma_{p,d}}{1+exp(-\beta_{p,d}([Ca]-\theta_{p,d}))},$$ (2)

where *γ*_*p*_>*γ*_*d*_ are potentiation and depression rates, *β*_*p*,*d*_ are the slopes at potentiation and depression boundaries *θ*_*p*_>*θ*_*d*_ (Fig 1A). The depression rate is smaller than the potentiation rate for provoking a transition from depressed to potentiated state for a high probability [29,30,32]. We followed [29] for the value of slopes. We studied the impact of the boundaries on the long-term plasticity in the manuscript.

The calcium concentration is controlled by the NMDAR-mediated current $I_{NMDA}^{\left[Ca\right]}$:

$$\frac{d[Ca]}{dt}=I_{NMDA}^{\left[Ca\right]}-\frac{\left[Ca\right]}{\tau_{ca}},$$ (3)

where *τ*_*ca*_ is the decay time constant of calcium influx. The NMDAR-mediated calcium current $I_{NMDA}^{\left[Ca\right]}$ depend on both the presynaptic activity via the kinetics of glutamate binding and the postsynaptic membrane potential [29] expressed as,

$$I_{NMDA}^{\left[Ca\right]}=G(F_{pre})h_{ca}H(V_{post}),$$ (4)

where *G*(*F*_*pre*_) formulates the kinetics of the glutamate binding depending on the firing rate of the presynaptic population, *F*_*pre*_. The function *H*(*V*_*post*_) (Fig 1B) formulates the relation between the NMDA currents and the postsynaptic (population level) depolarization:

$$H(V_{post})=\frac{1}{1+exp(-\mu (V_{post}-V_{th}))},$$ (5)

where *μ* is the slope at *V*_*th*_ = *V*_*post*_. The function *H*(*V*_*post*_) can be interpreted as the magnesium blockage at the population level. Parameter *h*_*ca*_ is a scaling factor, which distinguishes the NMDAR-mediated postsynaptic depolarization from calcium entry.

Plasticity of excitatory synapses is modeled at both presynaptic and postsynaptic levels. Presynaptic plasticity corresponds to variations in glutamate release probability, running on short- and long-timescales. The presynaptic STP rule follows [41]:

$$\frac{dr}{dt}=\frac{1-r}{\tau_{r}}-u\;r\;F_{pre}$$ (6A)

$$\frac{du}{dt}=\frac{U_{s}-u}{\tau_{f}}+U_{s}(1-u)F_{pre}$$ (6B)

where *r* is the amount of available neurotransmitters, *u* is the utilization factor, *τ*_*r*_ is the recovery timescale, *τ*_*f*_ is the facilitation timescale and *U*_*s*_ is the neurotransmitter release probability. Since the interaction between glutamatergic synapses is depression dominated [42,43], we consider *τ*_*r*_>*τ*_*f*_.

The presynaptic long-term plasticity is the long-term variation in the neurotransmitter release probability *U*_*s*_ controlled by the calcium-dependent retrograde signaling [30],

$$\frac{dU_{s}}{dt}=\frac{1}{\tau_{U}}(U_{s}^{d}-U_{s}+\rho (U_{s}^{p}-U_{s}^{d})).$$ (7)

Parameters $U_{s}^{d}$ and $U_{s}^{p}$ represent the depressed and potentiated states of the release probability *U*_*s*_, and *τ*_*U*_ is the time constant of the presynaptic long-term plasticity.

Postsynaptic long-term plasticity corresponds to an insertion/removal of glutamatergic AMPAR,

$$\frac{d{\tilde{C}}_{AMPA}}{dt}=\frac{1}{\tau_{C_{AMPA}}}(C_{AMPA}^{d}-{\tilde{C}}_{AMPA}+\rho (C_{AMPA}^{p}-C_{AMPA}^{d})).$$ (8)

Parameters $C_{AMPA}^{d}$ and $C_{AMPA}^{p}$ represent the depressed and potentiated states of the AMPAergic coupling strength ${\tilde{C}}_{AMPA}(t)$, and $\tau_{C_{AMPA}}$ is the time-constant of the postsynaptic long-term plasticity. For simplicity, we assume that presynaptic and postsynaptic long-term changes have the same time constants $\tau_{U}=\tau_{C_{AMPA}}$. Since the expression of synaptic plasticity is slower than its induction [44], we consider $\tau_{p}<\tau_{U}=\tau_{C_{AMPA}}$ as in [30].

The whole model of pre- and postsynaptic plasticity reads as follows:

$$\frac{dr}{dt}=\frac{1-r}{\tau_{r}}-u\;r\;F_{pre},$$ (9A)

$$\frac{du}{dt}=\frac{U_{s}-u}{\tau_{f}}+U_{s}(1-u)F_{pre},$$ (9B)

$$\frac{d[Ca]}{dt}=I_{NMDA}^{\left[Ca\right]}-\frac{\left[Ca\right]}{\tau_{ca}},$$ (9C)

$$\frac{d\rho }{dt}=\frac{1}{\tau_{\rho }}(-\rho (1-\rho )(\rho_{*}-\rho )+(1-\rho )\Omega_{p}([Ca])-\rho \Omega_{d}([Ca])),$$ (9D)

$$\frac{dU_{s}}{dt}=\frac{1}{\tau_{U}}(U_{s}^{d}-U_{s}+\rho (U_{s}^{p}-U_{s}^{d})),$$ (9E)

$$\frac{{d\tilde{C}}_{AMPA}}{dt}=\frac{1}{\tau_{C_{AMPA}}}(C_{AMPA}^{d}-{\tilde{C}}_{AMPA}+\rho (C_{AMPA}^{p}-C_{AMPA}^{d})).$$ (9F)

The parameter values of (9) are given in Table 1. Except the values of time constants as mentioned above, the parameter values are chosen in accordance with the dynamics of the interacting NMMs detailed in the following sections.

**Table 1:** Variables and parameter values of (9) unless otherwise stated.

	Variables	Equation number
*r*(*t*)	amount of available neurotransmitters	Eq 9A
*u*(*t*)	utilization factor of neurotransmitters	Eq 9B
[*Ca*](*t*)	calcium concentration	Eq 9C
*ρ*(*t*)	state of the synaptic efficacy	Eq 9D
${\tilde{C}}_{AMPA}(t)$	AMPAergic coupling strength	Eq 9E
*U*_*s*_(*t*)	neurotransmitter release probability	Eq 9F
	Parameters	Value
*θ*_*d*_	depression threshold	0.1
*θ*_*p*_	potentiation threshold	0.4
*γ*_*d*_	depression amplitude	1
*γ*_*p*_	potentiation amplitude	5
*β*_*d*_	depression slope	80
*β*_*p*_	potentiation slope	80
*μ*	slope of NMDA-dependent postsynaptic depolarization	1
*V*_*th*_	threshold of NMDA-dependent postsynaptic depolarization	5 V
*h*_*ca*_	calcium current scaling factor	10
*τ*_*d*_	presynaptic short-term depression time constant	0.200 sec
*τ*_*f*_	presynaptic short-term facilitation time constant	0.05 sec
*τ*_*ca*_	the calcium influx decay time constant	0.05 sec
*τ*_*ρ*_	synaptic efficacy time constant	50 sec
*τ*_*U*_	presynaptic long-term plasticity time constant	100 sec
$\tau_{C_{AMPA}}$	postsynaptic long-term plasticity time constant	100 sec
$U_{s}^{d}$	Value of depressed release probability	0.4
$U_{s}^{p}$	Value of potentiated release probability	0.8
$C_{AMPA}^{d}$	Value of depressed AMPAergic coupling strength	50
$C_{AMPA}^{p}$	Value of potentiated AMPAergic coupling strength	100

### The revisited neural mass model

**Fig 2:**
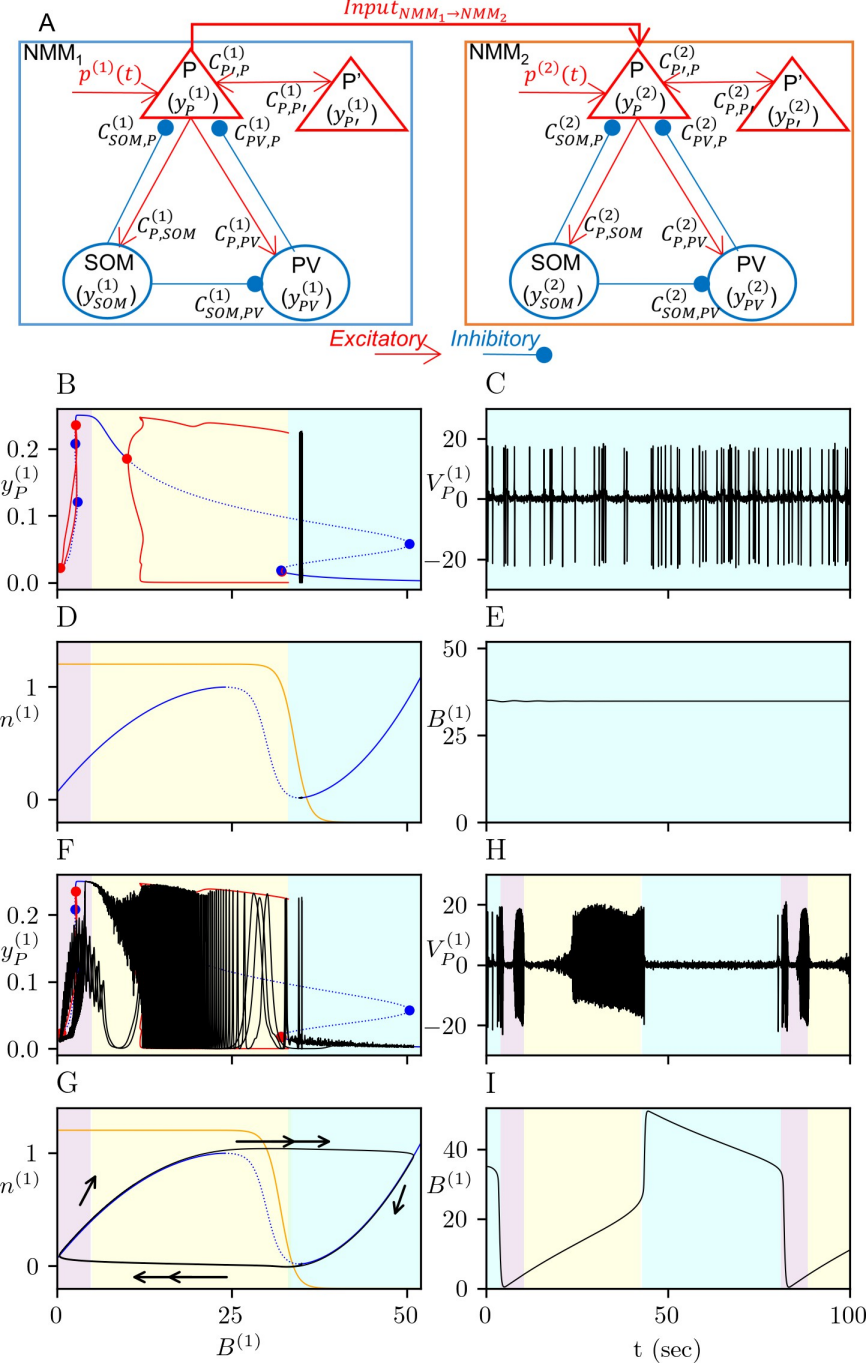
Unidirectionally interacting NMM_1_ and NMM_2_ and the dynamics of NMM_1_ in the interictal and ictal regimes. (A) Model diagram of unidirectionaly coupled NMM_1_ and NMM_2_. Each NMM includes four interacting subpopulations of pyramidal neurons (P and P’) and inhibitory interneurons (PV and SOM) with excitatory (red connections) and inhibitory (blue connections) interactions between parametrized by the coupling coefficients $C_{k,p}^{\left(i\right)}$ for *i*∈{1,2} with *k* and *p* representing the pre- and postsynaptic subpopulations of NMM_i_, respectively. The PSP generated by a subpopulation *k* are denoted by $y_{k}^{\left(i\right)}$. NMM_2_ is subject to an excitatory input from NMM_1_, denoted by $Input_{NMM_{1}\to NMM_{2}}$. (B-E) Dynamics of of (10)-(11) for NMM_1_ in the interictal regime for $b_{thr}^{\left(1\right)}=34$. (B) Bifurcation diagram of (10) where the amplitude of $y_{P}^{\left(1\right)}$ is presented as a function of *B*^(1)^. The blue curve shows the branch of equilibrium points (bold for stable and dashed for unstable equilibrium points). The red curves show the amplitude of $y_{P}^{\left(1\right)}$ in the oscillatory regime. The Hopf bifurcations along the branch of equilibrium points are denoted by red dots and saddle-node bifurcation by blue dots. The range of *B*^(1)^ values that corresponds to the fast onset, ictal and interictal periods are marked by purple, yellow and cyan patches, respectively. The time solution (black curve) is superimposed on the bifurcation diagram. (C) Time trace for $V_{P}^{\left(1\right)}$ showing interictal spikes. (D) Phase plane of (11) with the *B*^(1)^-nullcline (blue curve, bold for stable and dashed for branches) and the *n*^(1)^-nullcline (orange curve). The range of *B*^(1)^ values that corresponds to the fast onset, ictal and interictal periods in (10) are marked by green, yellow and cyan patches, respectively. The time solution (black curve) is superimposed on the bifurcation diagram. (E) Time trace for *B*^(1)^. (F-I) Dynamics of (10)-(11) for NMM_i_ in the ictal regime for $b_{thr}^{\left(1\right)}=32$. Same color codes and markers in (B-E) is used. Arrows in panel (G) indicates the direction of the trajectory (black curve) on the (*B*^(1)^,*n*^(1)^)-plane with double arrows indicating the fast transitions. The fast onset, ictal and interictal periods are marked on the time traces of $V_{P}^{\left(1\right)}$ and *B*^(1)^ on panels H and I, respectively.

We revisited a minimal NMM of the CA1 region of the hippocampus [40] for obtaining an autonomous model that can undergoes seizures for a fixed parameter set. The original model includes four interacting neuronal subpopulations: two interconnected subpopulations of glutamatergic pyramidal neurons (P, P’), and two subpopulations of GABAergic inhibitory interneurons (somatostatin positive (SOM), and parvalbunim positive (PV), also called dendrite-projecting slow and soma-projecting fast interneurons, respectively). The activity of each subpopulation is described by a “wave to pulse” function, *S*(*v*) = 5/(1+exp(0.56(6−*v*))), transforming the net membrane polarization in response to synaptic inputs into a firing rate. The synaptic interactions between the subpopulations is described by a “pulse to wave” that converts the input average firing rate into a postsynaptic potential (PSP), which can be either excitatory–EPSP—or inhibitory—IPSP) at the input of each subpopulation, that is *h*(*t*) = *W*/*τ*_*w*_ *t* exp(−*t*/*τ*_*w*_), where W represents the average PSP amplitude and *τ*_*w*_ is the time constant. This linear filter, also known as the alpha-function, introduces a second order ordinary differential equation $\frac{d^{2}y}{{dt}^{2}}=\frac{W}{\tau_{w}}S(v)-\frac{2}{\tau_{w}}\frac{dy}{dt}-\frac{1}{\tau_{w}^{2}}y$ where *y*(*t*) is the PSP in response to the input *S*(*v*). The equations of an unconnected population *i* NMM_i_ reads:

$$\frac{{d^{2}y}_{P}^{\left(i\right)}}{{dt}^{2}}=\frac{A^{\left(i\right)}}{\tau_{a}}S(V_{P}^{\left(i\right)})-\frac{2}{\tau_{a}}\frac{{dy}_{P}^{\left(i\right)}}{dt}-\frac{1}{\tau_{a}^{2}}y_{p}^{\left(i\right)},$$ (10A)

$$\frac{{d^{2}y}_{P^{\prime }}^{\left(i\right)}}{{dt}^{2}}=\frac{A^{\left(i\right)}}{\tau_{a}}(p^{\left(i\right)}(t)+C_{P^{\prime },P}^{\left(i\right)}S({C_{P,P^{'}}^{\left(i\right)}y}_{P}^{\left(i\right)}))-\frac{2}{\tau_{a}}\frac{{dy}_{P^{\prime }}^{\left(i\right)}}{dt}-\frac{1}{\tau_{a}^{2}}y_{P^{\prime }}^{\left(i\right)},$$ (10B)

$$\frac{{d^{2}y}_{SOM}^{\left(i\right)}}{{dt}^{2}}=\frac{B^{\left(i\right)}}{\tau_{b}}S({C_{P,SOM}^{\left(i\right)}y}_{P}^{\left(i\right)})-\frac{2}{\tau_{b}}\frac{{dy}_{SOM}^{\left(i\right)}}{dt}-\frac{1}{\tau_{b}^{2}}y_{SOM}^{\left(i\right)},$$ (10C)

$$\frac{{d^{2}y}_{PV}^{\left(i\right)}}{{dt}^{2}}=\frac{G^{\left(i\right)}}{\tau_{g}}S({C_{P,PV}^{\left(i\right)}y}_{P}^{\left(i\right)}-{C_{SOM,PV}^{\left(i\right)}y}_{SOM}^{\left(i\right)})-\frac{2}{\tau_{g}}\frac{{dy}_{PV}^{\left(i\right)}}{dt}-\frac{1}{\tau_{g}^{2}}y_{PV}^{\left(i\right)},$$ (10D)

with variables $y_{k}^{\left(i\right)}$ representing the PSP generated by a subpopulation *k*. Parameters $C_{k,p}^{\left(i\right)}$ scales the impact from subpopulation *k* to subpopulation *p* within the population NMM_i_. The net polarization of P subpopulation of NMM_i_, $V_{P}^{\left(i\right)}$, reads,

$$V_{P}^{\left(1\right)}=\Sigma (C_{k,P}^{\left(1\right)}y_{k}^{\left(1\right)})$$

for NMM_1,_ and,

$$V_{P}^{\left(2\right)}=\Sigma (C_{k,P}^{\left(2\right)}y_{k}^{\left(2\right)})+\Sigma Input_{NMM_{1}\to NMM_{2}}$$

for NMM_2_, which is subject to the excitatory AMRAergic and NMDAergic signals from NMM_1_ on its P subpopulation, $\Sigma Input_{NMM_{1}\to NMM_{2}}$. We consider $V_{P}^{\left(i\right)}$ as the model output, which is a simplified proxy for the local field potential recorded by an extracelluar electrode positioned in the neuronal population. The model diagram is given in Fig 2A.

Intracranial recordings from epileptic patients with focal epilepsy show four distinct phases of activity: interictal phase with sporadic spikes, preictal phase with periodic preictal spikes, tonic low-voltage fast onset marked by a gamma-band activity, and clonic discharges [45]. Recordings show sequential transitions between these phases, that is, interictal, preictal, then, ictal phase (so-called *seizure*, which starts with a tonic low-voltage fast onset phase followed by clonic discharges). For an appropriate choice of model parameters, the transitions between these phases in (10) can be obtained by varying the IPSP amplitude of the SOM interneurons, B^(i)^, (e.g. [40,46]). Fig 2B shows the bifurcation diagram of (10) for NMM_1_ as a function of *B*^(1)^ with the parameter set in Table 2. The branch of equilibrium points undergoes four Hopf bifurcations at *B*^(1)^≈{0.47,2.66,9.98,32.14} and saddle-node bifurcations at *B*^(1)^≈{2.60,2.92,32.01,50.38}. The gamma-band oscillations lie in *B*^(1)^≈(2.66,0.47) and the tonic lower frequency oscillations in *B*^(1)^≈(9.98,32.14). Under a stochastic input, such as $p^{\left(i\right)}(t)=N(p_{m}^{\left(i\right)},p_{S})$ in (10B), the gamma-band oscillations can be observed for 0<*B*^(1)^<4, the tonic phase for 4<*B*^(1)^<32. The low-frequency periodic preictal spikes and aperiodic interictal spikes can be located at the boundary between tonic oscillations and stable equilibrium points for 32<*B*^(1)^<50.

**Table 2:** Variables and parameters values for (10)-(15) unless otherwise stated.

	Variables of (10)	Equation number	
$y_{P}^{\left(i\right)}(t)$	EPSP emitted by the subpopulation P	Eq 10A	
$y_{P'}^{\left(i\right)}(t)$	EPSP emitted by the subpopulation P’	Eq 10B	
$y_{SOM}^{\left(i\right)}(t)$	IPSP emitted by the subpopulation SOM	Eq 10C	
$y_{PV}^{\left(i\right)}(t)$	IPSP emitted by the subpopulation PV	Eq 10D	
	Parameters of (10)	NMM_1_	NMM_2_
*A* ^(*i*)^	EPSP amplitude [mV]	5	5
*G* ^(*i*)^	IPSP_PV_ amplitude [mV]	20	2
*τ*_*a*_	EPSP time constant [s]	0.01	0.01
*τ*_*b*_	IPSP_SOM_ time constant [s]	0.03	0.03
*τ*_*g*_	IPSP_PV_ time constant [s]	0.003	0.003
$C_{P,P^{'}}^{\left(i\right)}$	*P → P*^*′*^ coupling coefficient	135	135
$C_{P^{\prime },P}^{\left(i\right)}$	*P*^*′*^ *→ P* coupling coefficient	108	108
$C_{SOM,P}^{\left(i\right)}$	*SOM → P* coupling coefficient	35	35
$C_{PV,P}^{\left(i\right)}$	*PV → P* coupling coefficient	200	200
$C_{P,SOM}^{\left(i\right)}$	*P → SOM* coupling coefficient	25	25
$C_{P,PV}^{\left(i\right)}$	*P → PV* coupling coefficient	200	200
$C_{SOM,PV}^{\left(i\right)}$	*SOM → PV* coupling coefficient	120	120
$p_{m}^{\left(i\right)}$	mean of the Gaussian input *p*^(*i*)^(*t*)	90	70
$p_{s}^{\left(i\right)}$	standard deviation of the Gaussian input *p*^(*i*)^(*t*)	2	2
	Variables of (11)	Equation number	
*B*^(*i*)^(*t*)	IPSP_SOM_ amplitude [mV]	Eq 11A	
*n*^(*i*)^(*t*)	Auxiliary variable	Eq 11B	
	Parameters of (11)	NMM_1_	NMM_2_
*p*_1_	local maximum of the *B*^(*i*)^-nullcline	25	25
*p*_2_	inflection point of the middle branch of the *B*^(*i*)^-nullcline	30	30
*p*_3_	local minimum of the *B*^(*i*)^-nullcline	33	33
*m*_1_	slope of the of the left branch of *B*^(*i*)^-nullcline	0.0015	0.0015
*m*_3_	slope of the of the right branch of the *B*^(*i*)^-nullcline	0.003	0.003
*n*_*k*_	minimum of the *n*-nullcline	-0.2	-0.2
*n*_*p*_	maximum of the *n*-nullcline sigmoid	1.4	1.4
*n*_*r*_	slope of the *n*-nullcline at the inflection point	2	2
$b_{thr}^{\left(i\right)}$	inflection point of the *n*-nullcline	34	44
*δ*	timescale parameter	50	50
*ε*	timescale parameter	0.05	0.05
	Variables of (12)-(15)	Equation number	
*y*_*AMPA*_(*t*)	AMPAergic-EPSP emitted by the input from NMM_1_ onto NMM_2_	Eq 12	
*y*_*NMDA*_(*t*)	NMDAergic-EPSP emitted by the input from NMM_1_ onto NMM_2_	Eq 13	
*y*_*NMDA*,*ext*_(*t*)	NMDAergic-EPSP emitted by the activation of extrasynaptic NMDAR	Eq 14	
*K*(*t*)	Auxiliary variable representing the changes in GABAergic activity	Eq 15	
	Parameter values of (12)-(15)	NMM_1_	NMM_2_
$A_{AMPA}^{\left(2\right)}$	AMPAergic-EPSP amplitude [mV]	-	10
$A_{NMDA}^{\left(2\right)}$	NMDAergic-EPSP amplitude [mV]	-	2
$A_{NMDA,ext}^{\left(2\right)}$	Extrasynaptic NMDAergic-EPSP amplitude [mV]	-	1
*τ*_*AMPA*_	AMPAergic-EPSP time constant [s]	-	0.005
*τ*_*NMDA*_	NMDAergic-EPSP time constant [s]	-	0.02
*τ*_*NMDA*,*ext*_	Extrasynaptic NMDAergic-EPSP time constant [s]	-	0.04
*C*_*NMDA*_	NMDAergic coupling coefficient	-	*C*_*AMPA*,*min*_
*τ*_*K*_	Extrasynaptic NMDA integration time constant	-	10

One can ensure an autonomous transition between the phases of epileptic activity by modeling *B*^(*i*)^ as a variable that varies in the range where these phases are observed. Here, we introduce a slow-subsystem where the IPSP amplitude of the SOM interneurons *B*^(*i*)^ is a variable:

$$\frac{{dB}^{(i)}}{dt}=\delta (n^{\left(i\right)}-(-\frac{m_{1}{\left(B^{\left(i\right)}-p_{1}\right)}^{2}}{1+\exp\left(-p_{1}+B^{\left(i\right)}\right)}+\frac{1}{1+\exp\left(-p_{2}+B^{\left(i\right)}\right)}+\frac{m_{3}{\left(B^{\left(i\right)}-p_{3}\right)}^{2}}{1+\exp\left(-p_{3}+B^{\left(i\right)}\right)})),$$ (11A)

$$\frac{dn^{\left(i\right)}}{dt}=\varepsilon \left(-n^{\left(i\right)}+n_{k}+\frac{n_{p}}{1+\exp\left(-n_{r}(b_{thr}^{\left(i\right)}-B^{\left(i\right)})\right)}\right).$$ (11B)

Parameters of the *B*^(*i*)^− and *n*^(*i*)^-nullclines can be adjusted with respect to the critical (bifurcation) points of (10), desired type and range of behavior. Among these parameters, *p*_1_ and *p*_3_ correspond to the jump points in the *B*^(*i*)^-space from ictal to interictal and from interictal to ictal, respectively. Parameters *m*_1_ and *m*_3_ control the stiffness of the left and right branches of the *B*^(*i*)^-nullcline. Parameter *m*_1_ is crucial for covering the different phases of the ictal regime starting with gamma-band oscillations and continuing by tonic spiking. Parameter *m*_3_ contributes to the duration of the silent phase following the jump from ictal to interictal phase. Parameters *m*_1_,*p*_1_,*δ* and *ϵ* together determine the duration of the ictal phase. The parameter $b_{thr}^{\left(i\right)}$ is the main parameter controlling the excitability, hence, the susceptibility of seizing of NMM_i_. Parameter values of (11) are given in Table 2.

Note that the system (11) is a slow-fast system itself, where *B*^(*i*)^ is the fast and *n*^(*i*)^ is the slow variable. The *B*^(*i*)^-nullcline of (11) has an N-shape with stable outer and unstable middle branches (Fig 2D). System (10) governs the interictal and preictal phases for the *B*^(*i*)^ values on the right branch *B*^(*i*)^-nullcline (Fig 2B–2E). The *B*^(*i*)^ values on the left and middle branches *B*^(*i*)^-nullcline correspond to the ictal phase (fast-onset and tonic discharges) of (10). Depending on the position of the equilibrium point of (11), defined as the intersection between the N-shaped *B*^(*i*)^-nullcline and sigmoidal *n*^(*i*)^-nullcline, the system (11) follows either a steady state (Fig 2D) or an oscillatory solution (Fig 2G). If the stable equilibrium point is close to the right fold point of the *B*^(*i*)^-nullcline, then (11) is in an excitable state, i.e any perturbation on *B*^(*i*)^ can trigger a jump to the left branch of the fast nullcline, which corresponds to the ictal regime starting with a fast-onset activity. As the trajectory of (11) moves along the left branch of the *B*^(*i*)^-nullcline, the system (10) exhibits the fast-onset, then tonic spiking before jumping back to the right branch, that is the interictal regime (Fig 2F–2I).

Table 2 shows three main differences between the parameters of uncoupled NMM_1_ and NMM_2_. The first difference concerns the mean values of the external inputs ($p_{m}^{\left(i\right)}$), which determines the range of the IPSP amplitude of the SOM interneurons (*B*^(*i*)^(*t*)) for which sporadic and tonic spiking are observed in the model. The second difference is the IPSP amplitude of the PV interneurons (*G*^(*i*)^), where higher values give gamma-band activity in the model. The third difference is the excitability ($b_{thr}^{\left(i\right)}$), which controls both *B*^(*i*)^(*t*) and the susceptibility of an NMM to jump from the interictal phase to seizure. The lower $b_{thr}^{\left(i\right)}$, the more susceptible the NMM is to exhibiting a seizure. For simplicity, we considered the same values for the rest of the parameters. The bifurcation diagram of uncoupled NMM_2_ and its response to the epileptic activity when coupled to NMM_1_ (Fig 2) are given in S1 and S2 Figs.

### Modeling plasticity with coupled neural mass models

We consider two populations NMM_1_ and NMM_2_ modelled by the NMM formulation given in (10)-(11). NMM_2_ receives excitatory inputs from NMM_1_ through AMPAergic and NMDAergic signaling. Let $F_{P}^{\left(1\right)}$ be the firing rate of the P subpopulation of the NMM_1_, *r*^(*i*)^(*t*) and *u*^(*i*)^(*t*) are the variables describing the presynaptic neurotransmitter release (6). Then the AMPAergic EPSP is given by

$$\frac{{d^{2}y}_{AMPA}}{d^{2}t}=\frac{r^{\left(1\right)}u^{\left(1\right)}A_{AMPA}^{\left(2\right)}}{\tau_{AMPA}}F_{P}^{\left(1\right)}-\frac{2}{\tau_{AMPA}}\frac{{dy}_{AMPA}}{dt}-\frac{1}{\tau_{AMPA}^{2}}y_{AMPA},$$ (12)

where *τ*_*AMPA*_ is the synaptic time constant and $A_{AMPA}^{\left(2\right)}$ is the amplitude that is modulated by STP variables *r*^(1)^(*t*) and *u*^(1)^(t). The NMDAergic-EPSP is given by

$$\frac{{d^{2}y}_{NMDA}}{d^{2}t}=\frac{r^{\left(1\right)}u^{\left(1\right)}A_{NMDA}^{\left(2\right)}}{\tau_{NMDA}}F_{P}^{\left(1\right)}-\frac{2}{\tau_{NMDA}}\frac{{dy}_{NMDA}}{dt}-\frac{1}{\tau_{NMDA}^{2}}y_{NMDA},$$ (13)

where *τ*_*NMDA*_ is the synaptic time constant and $A_{NMDA}^{\left(2\right)}$ is the amplitude. The NMDAergic EPSP is slower than the AMPAergic EPSP [47], thus *τ*_*AMPA*_<*τ*_*NMDA*_. The net depolarization of the postsynaptic subpopulation due to the activation of NMDAR depends on the postsynaptic potential, $V_{P}^{\left(2\right)}$ as well as the presynaptic entry *C*_*NMDA*_*y*_*NMDA*_, i.e. $C_{NMDA}y_{NMDA}H(V_{P}^{\left(2\right)})$, with *C*_*NMDA*_ is the coupling strength.

The pathological plasticity is activated when both the probability of vesicle opening *U*_*s*_ and the AMPAR insertion ${\tilde{C}}_{AMPA}$ are potentiated (9). Under this condition, an afferent epileptic spike from NMM_1_, which releases high levels of glutamate, activates the extrasynaptic NMDAR. The EPSP mediated by extrasynaptic NMDR, *y*_*NMDA*,*ext*_, has slower kinetics than the EPSP mediated by synaptic NMDAR [48] (i.e. *τ*_*NMDA*_<*τ*_*NMDA*,*ext*_) and *y*_*NMDA*,*ext*_ is given by

$$\frac{{d^{2}y}_{NMDA,ext}}{d^{2}t}=\frac{r^{\left(1\right)}u^{\left(1\right)}A_{NMDA,ext}^{\left(2\right)}}{\tau_{NMDA,ext}}F_{P}^{\left(1\right)}-\frac{2}{\tau_{NMDA,ext}}\frac{{dy}_{NMDA,ext}}{dt}-\frac{1}{\tau_{NMDA,ext}^{2}}y_{NMDA,ext},$$ (14)

with the postsynaptic depolarization $C_{NMDA}y_{NMDA,ext}H(V_{P}^{\left(2\right)})$. The net postsynaptic polarization of the P subpopulation of the NMM_2_ then reads,

$$V_{P}^{\left(2\right)}=\Sigma (C_{k,P}^{\left(2\right)}y_{k}^{\left(2\right)})+{\tilde{C}}_{AMPA}y_{AMPA}+C_{NMDA}{(y}_{NMDA}H(V_{P}^{\left(2\right)})+y_{NMDA,ext}H(V_{P}^{\left(2\right)}))$$

with the plastic ${\tilde{C}}_{AMPA}(t)$ given in (9) and constant *C*_*NMDA*_.

The changes in GABAergic activity caused by the activation of extrasynaptic NMDAR is modeled via an auxiliary variable *K*(*t*),

$$\frac{dK}{dt}=\frac{1}{\tau_{K}}\left(-K(0.5-K)(1-K)-C_{NMDA}y_{NMDA,ext}H(V_{P}^{\left(2\right)})\right).$$ (15)

When extrasynaptic NMDAR is inactive (i.e. *y*_*NMDA*,*ext*_ = 0), the Eq 15 has three equilibrium points at K* = {0,0.5,1}. The equilibria at K* = {0,1} are stable, but the equilibrium at K* = 0.5 is unstable. The extrasynaptic NMDAR activation (i.e. *y*_*NMDA*,*ext*_≠0) can lead to a saddle-node bifurcation of the equilibria K* = 0.5 and K* = 1 leaving (15) with a single equilibrium point at K*≈0, which becomes the global attractor of (15). Inactivation of the extrasynaptic NMDAR reintroduces the three equilibria but the transition from *K*≈0 to *K*≈1 state would not be possible in the model unless *y*_*NMDA*,*ext*_ is negative. The latter is not biologically plausible or computationally possible because NMDA-mediated currents are excitatory. Therefore, the transition from *K*≈0 to *K*≈1 is irreversible. This phenomenological formulation is based on experimental studies in animal models of epilepsy, which suggest that when the secondary focus “matures”, abolition of the primary focus or disruption of cortical connections does not abolish the secondary epileptic zone [27,49].

The variable *K*(*t*) modulates the excitability of NMM_2_ as,

$$b_{thr}^{\left(2\right)}(t)=b_{thr}^{\left(2\right)}-k_{B}^{\left(2\right)}(1-K(t)),$$

where the parameter $k_{B}^{\left(2\right)}$ scales the impact of *K*(*t*) on the excitability. Since the gamma-band activity observed at the seizure onset can be obtained by increasing the IPSP amplitude of PV interneurons [40,46], *K*(*t*) modulates *G*^(2)^ as

$$G^{\left(2\right)}(t)=G^{\left(2\right)}+k_{G}^{\left(2\right)}(1-K(t)),$$

where the parameter $k_{G}^{\left(2\right)}$ scales the impact of *K*(*t*) on the IPSP amplitude. We assumed that, in the absence of extrasynaptic NMDAR activity, *K*(*t*) = 1, for which IPSP amplitude is too small for yielding a gamma-band activity in (10) and the equilibrium point of (11) is far from the critical point of the seizure transition (i.e. the *n*^(2)^-nullcline intersects the *B*^(2)^-nullcline on its right branch). As *K*(*t*)→0 with the activation of the extrasynaptic NMDAR, *G*^(2)^(*t*) increases to a suitable level for obtaining gamma-band activity, whereas $b_{thr}^{\left(2\right)}(t)$ decreases and drives the equilibrium point of (10) towards the critical point of seizure transition in (10)-(11). In other words, how far (11) is from a Hopf bifurcation, hence how far (10) is from seizing, is controlled by the extrasynaptic NMDAR activity.

The parameter values of Eqs 12–15 are given in Table 2. We followed the order of magnitude between different receptor types [47,48]. The amplitude of PSPs and coupling strengths are chosen to achieve self-consistency. Stochastic differential equations were iterated using Euler-Maruyama method with a step size *dt* = 10e−5 second. Simulation files are available at https://github.com/elifkoksal/plasticNMM. Bifurcation analysis was done with AUTO-07p [50].

### Reduced system of physiological plasticity

The system of equations describing the physiological plasticity is a multiple timescale system with $(\tau_{d},\tau_{f})<\tau_{ca}<\tau_{\rho }<\tau_{U}=\tau_{C_{AMPA}}$ (Table 1). We benefit from the multiple timescale structure of (9) to reduce the complexity of the full model to a lower dimensional system of equations for analyzing the synaptic plasticity in the long term. We use the elements from singular perturbation theory to decompose the system into fast and slow subsystems and focus on the dynamic in the slow regime [51]. We assume that the slow regime is governed by the variables concerning LTD/LTP and consolidation, e.g. $\left(\rho ,U_{s},{\tilde{C}}_{AMPA}\right)$. In the slow regime, the fast variables describing STP, *r*^(1)^ and *u*^(1)^, are controlled by the slow variables, as:

$$r^{(1)s}=\frac{1}{1+\tau_{d}u^{(1)s}F_{P}^{\left(1\right)}},$$

$$u^{(1)s}=\frac{U_{s}(1+\tau_{f}F_{P}^{\left(1\right)})}{1+\tau_{f}U_{s}F_{P}^{\left(1\right)}}$$

where superscript (.)^*s*^ indicates the slow regime. The NMDA-mediated calcium dynamics in the slow regime is given by

$${\left[Ca\right]}^{s}=\tau_{ca}G(F_{P}^{\left(1\right)})h_{Ca}H(V_{P}^{\left(2\right)})=\tau_{ca}C_{NMDA}y_{NMDA}(t)h_{ca}H(V_{P}^{\left(2\right)}).$$

If the NMDAergic-EPSP *y*_*NMDA*_(*t*) is approximated by $y_{NMDA}\approx \tau_{NMDA}A_{NMDA}^{\left(2\right)}r^{(1)s}u^{(1)s}F_{P}^{\left(1\right)}$ and the AMPAergic-EPSP *y*_*AMPA*_(*t*) by, $y_{AMPA}\approx \tau_{AMPA}A_{AMPA}^{\left(2\right)}r^{(1)s}u^{(1)s}F_{P}^{\left(1\right)}$, the postsynaptic potential $V_{P}^{\left(2\right)}$ reads

$$V_{P}^{\left(2\right)}=\Sigma (C_{k,P}^{\left(2\right)}y_{k}^{\left(2\right)})+{\tilde{C}}_{AMPA}\tau_{AMPA}A_{AMPA}^{\left(2\right)}r^{(1)s}u^{(1)s}F_{P}^{\left(1\right)}+C_{NMDA}\tau_{NMDA}A_{NMDA}^{\left(2\right)}r^{(1)s}u^{(1)s}F_{P}^{\left(1\right)}.$$

For the NMDA-mediated calcium entry and postsynaptic depolarization to occur, the postsynaptic membrane should be sufficiently depolarized, i.e. $V_{P}^{\left(2\right)}>V_{th}$. This depolarization depends on the AMPAergic input. Assuming that the postsynaptic system is balanced for $F_{P}^{\left(1\right)}$ and intra-population interactions are independent of the external input, i.e. $\Sigma (C_{k,P}^{\left(2\right)}y_{k}^{\left(2\right)})=0$, the postsynaptic polarization in response to the AMPAergic input from NMM_1_ can be reduced to

$$V_{P}^{\left(2\right)}\approx {\tilde{C}}_{AMPA}\tau_{AMPA}A_{AMPA}^{\left(2\right)}r^{(1)s}u^{(1)s}F_{P}^{\left(1\right)},$$

and the calcium concentration to

$${{\left[Ca\right]}^{s}=C}_{NMDA}\tau_{NMDA}A_{NMDA}^{\left(2\right)}r^{(1)s}u^{(1)s}F_{P}^{\left(1\right)}h_{ca},ifV_{P}^{\left(2\right)}>V_{th},otherwise{\left[Ca\right]}^{s}\approx 0.$$

As the fast variables (*r*^(1)^,*u*^(1)^,[*Ca*]) are expressed as a function of the slow variables, ($\rho ,U_{s},{\tilde{C}}_{AMPA}$), the reduced (slow) system reads:

$$\frac{d\rho }{d\bar{t}}=-\rho (1-\rho )(\rho_{*}-\rho )+(1-\rho )\Omega_{p}([Ca])-\rho \Omega_{d}([Ca]),$$ (16A)

$$\frac{dU_{s}}{d\bar{t}}=\frac{\tau_{\rho }}{\tau_{U}}(U_{s}^{d}-U_{s}+\rho (U_{s}^{p}-U_{s}^{d})).$$ (16B)

$$\frac{d{\tilde{C}}_{AMPA}}{d\bar{t}}=\frac{\tau_{\rho }}{\tau_{C_{AMPA}}}(C_{AMPA}^{d}-{\tilde{C}}_{AMPA}+\rho (C_{AMPA}^{p}-C_{AMPA}^{d})),$$ (16C)

where $\bar{t}=\frac{t}{\tau_{\rho }}$. If $\frac{\tau_{\rho }}{\tau_{U}}=\frac{\tau_{\rho }}{\tau_{C_{AMPA}}}\ll 1$, then we can take them to zero limit and treat $\left(U_{s},{\tilde{C}}_{AMPA}\right)$ as parameters to investigate the variation of *ρ*.

## Results

### Physiological plasticity

**Fig 3:**
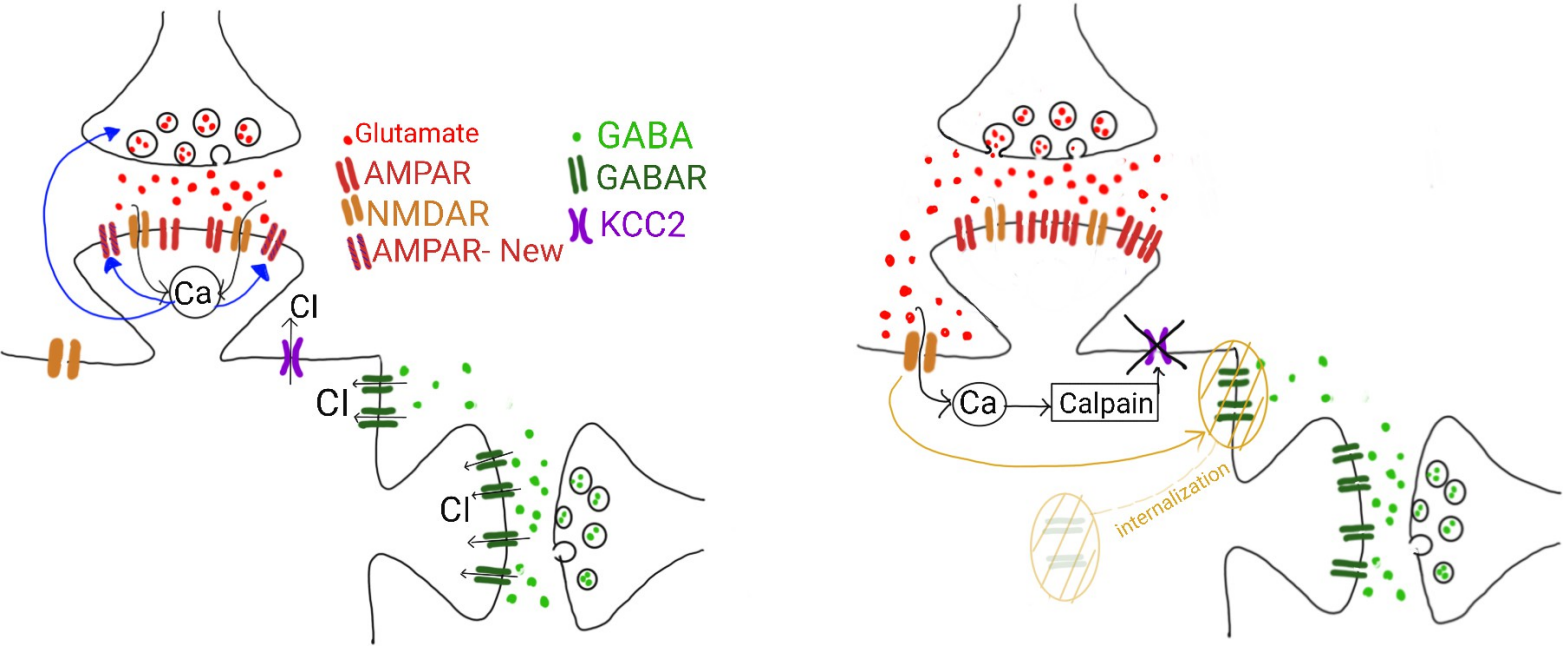
Physiological plasticity at presynaptic and postsynaptic sites. (A) Presynaptic glutamatergic release and subsequent postsynaptic depolarization results in calcium influx via NMDARs. Calcium signaling activates independent biochemical pathways, leading to postsynaptic and presynaptic long-term changes due to the insertion of new AMPARs and an increase in vesicle release probability. Under physiological conditions, excitation is balanced by sufficient chloride trafficking through GABAR and KCC2. (B) Pathological plasticity at postsynaptic site. When the released glutamate is too high to be used by synaptic AMPAR and NMDAR, it diffuses to the extrasynaptic site and activates the extrasynaptic NMDAR. Activation of NMDAR causes internalization of extrasynaptic GABAR. Calcium influx via the extrasynaptic NMDAR activates calpain that perturbs the KCC2 functioning.

**Fig 4:**
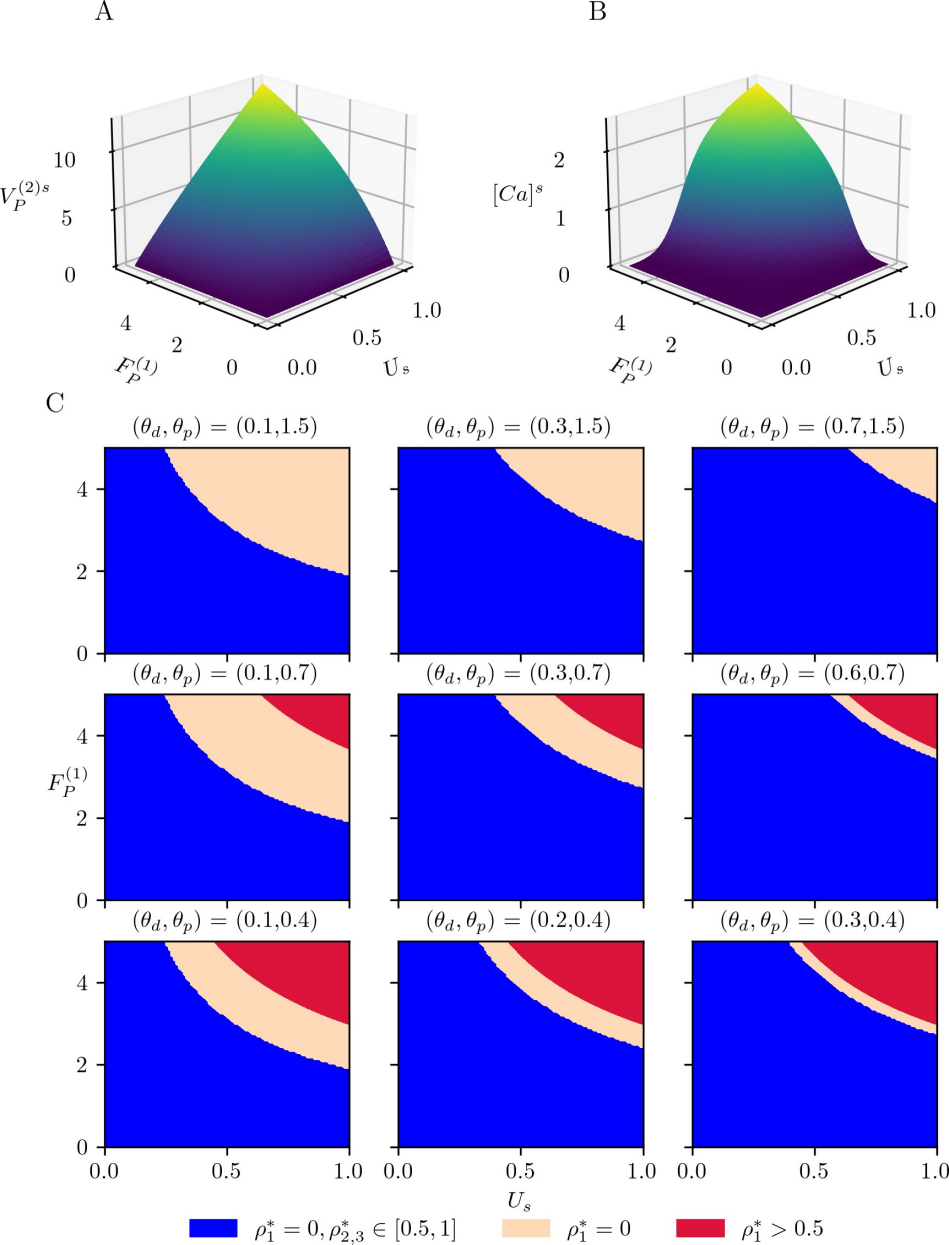
Postsynaptic depolarization, calcium concentrating and long-term plasticity in the slow regime. (A) Postsynaptic polarization $V_{P}^{\left(2\right)}$ and (B) calcium concentration [*Ca*]^*s*^ as a function of $\left(F_{P}^{\left(1\right)},U_{s}\right)$ for the parameter set given in Table 1 and *γ*_*p*_ = 2. (C) Equilibrium points of (16a) as function of $\left(F_{P}^{\left(1\right)},U_{s}\right)$ for different values of (*θ*_*p*_,*θ*_*d*_). In the blue regions (16a) has three equilibrium points at $\rho_{1,2,3}^{*}\in [0,1]$, a single equilibrium point at ρ* = 0 in the salmon regions, and a single equilibrium point at 0.5<ρ*≤1 in the red regions.

The model of physiological plasticity includes presynaptic STP, calcium-driven pre- and postsynaptic LTP/LTD and consolidation ((9), Fig 3A). These mechanisms run in different timescales, with STP being the fastest, and consolidation being the slowest. In Section Reduced system of physiological plasticity, we applied singular perturbation theory to obtain a reduced system of physiological plasticity. Here we assume that the timescales of calcium-driven long-term plasticity and consolidation are different enough ($\tau_{\rho }\ll \tau_{U}=\tau_{C_{AMPA}}$), to investigate the variation of *ρ*, which, in turn, controls the long-term variations in the neurotransmitter release probability, *U*_*s*_, and the AMPARergic coupling strength, ${\tilde{C}}_{AMPA}$, by treating these two slow variables as parameters. For a simple investigation of *ρ* (16a) for $\frac{\tau_{\rho }}{\tau_{U}}=\frac{\tau_{\rho }}{\tau_{C_{AMPA}}}=0$, we assume that ${\tilde{C}}_{AMPA}\approx C_{AMPA}^{d}+\kappa U_{s},(\kappa >0)$, which is a reasonable assumption since both ${\tilde{C}}_{AMPA}$ and *U*_*s*_ depend on *ρ* in the same manner. Fig 4A and 4B show $V_{P}^{\left(2\right)}$ and [*Ca*]^*s*^ as a function of $F_{P}^{\left(1\right)}$ and *U*_*s*_ with the parameter set given in Table 1. The postsynaptic subpopulation depolarizes monotonically for increasing $F_{P}^{\left(1\right)}$ and *U*_*s*_ (Fig 4A). The change in the calcium concentration follows a sigmoidal shape (Fig 4B). It remains close to zero for low values of *U*_*s*_ and $F_{P}^{\left(1\right)}$. It increases sharply for medium values of *U*_*s*_ and $F_{P}^{\left(1\right)}$, then slowly for higher values of *U*_*s*_ and $F_{P}^{\left(1\right)}$ as it approaches a plateau.

We then compute the equilibrium points of (16) for $\frac{dU_{s}}{d\bar{t}}=\frac{d{\tilde{C}}_{AMPA}}{d\bar{t}}=0$. In the absence of any pre- or postsynaptic activity, (16a) has three equilibrium points at ρ* = {0,0.5,1} with ρ* = {0,1} being stable and ρ* = 0.5 unstable. If the variations in *U*_*s*_ and $F_{P}^{\left(1\right)}$ are not sufficient to change the number of equilibrium points of (16A), the depressed synapses will remain depressed, and the potentiated ones will remain potentiated. Otherwise, (16A) has a single stable equilibrium point, such that, the synapses will either depress or potentiate. Fig 4C shows ρ* as a function of ($U_{s},F_{P}^{\left(1\right)}$) as the LTD and LTP thresholds, *θ*_*d*_ and *θ*_*p*_, vary on the calcium concentration surface (Fig 4B). The dark blue regions indicate that (16a) has three equilibrium points at ρ* = {0,0.5,1}, therefore, the synapses do not change. When the potentiation threshold *θ*_*p*_ is too high (*θ*_*p*_ =1.5, upper panels),the depressed state (salmon regions) is the only attractor for moderate to high input firing rates $F_{P}^{\left(1\right)}$ and release probability *U*_*s*_ depending on *θ*_*d*_. Lowering the potentiation threshold *θ*_*p*_ introduces a range of ($U_{s},F_{P}^{\left(1\right)}$) for which the potentiated state is an attractor (red regions in Fig 4C for *θ*_*p*_ = {0.4,0.7}. Consequently, both depressed and potentiated synapses are preserved for a low presynaptic activity. Lowering the potentiation threshold expands the range of LTP and, increasing *θ*_*d*_ shrinks the LTD range.

### Physiological plasticity under epileptic activity

**Fig 5:**
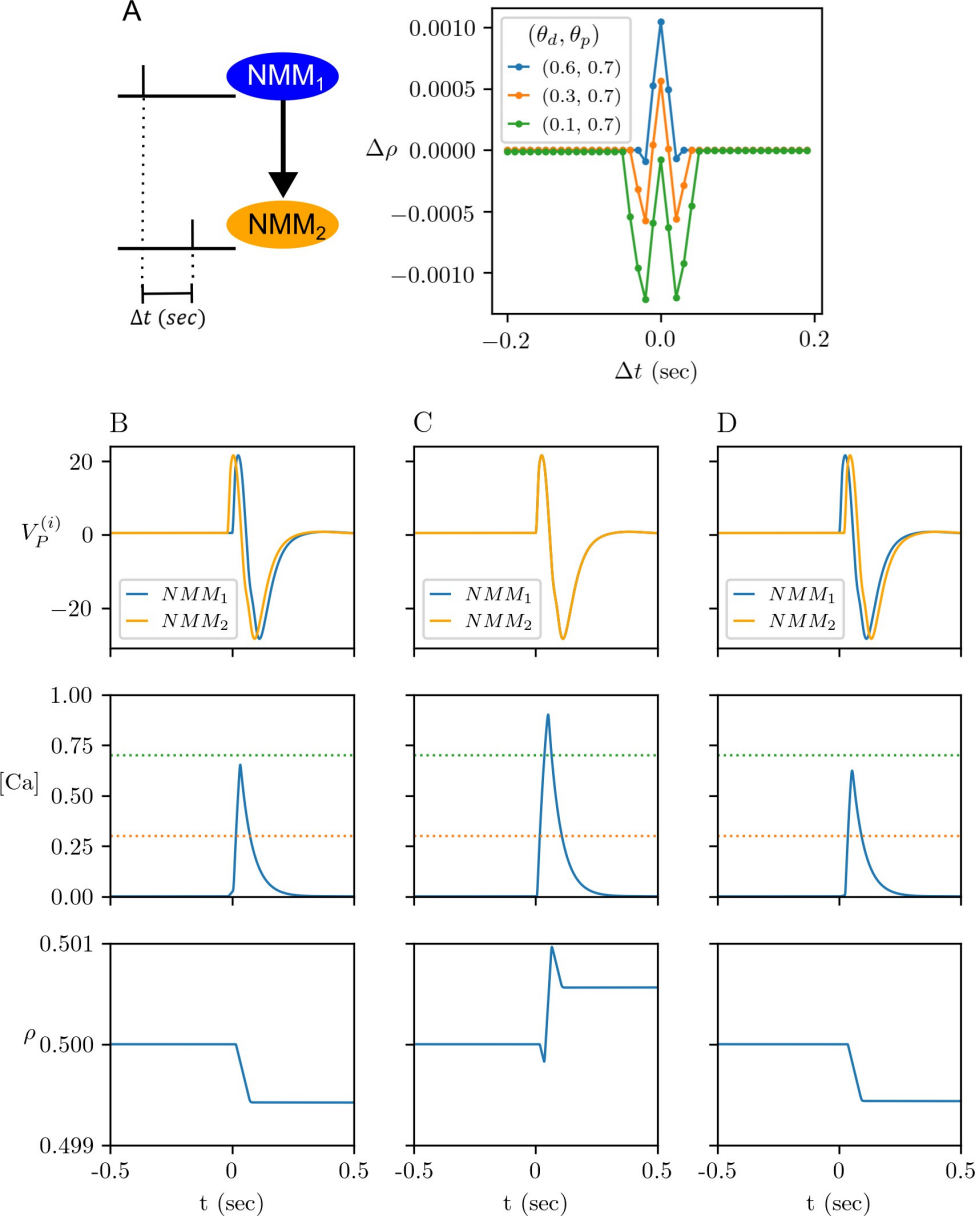
Change in synaptic efficacy under epileptic spikes. (A) Rate of change in the synaptic efficacy variable *ρ* under single epileptic spike in NMM_1_ and NMM_2_ when the system (9)-(11) is initialized from *ρ*(0) = 0.5 with $\left(U_{s},{\tilde{C}}_{AMPA})=(U_{s}^{d},C_{AMPA}^{d}\right)$. Epileptic spikes are triggered by a pulse stimulation applied with a time delay of Δ*t* (sec) for (*θ*_*d*_,*θ*_*p*_) = (0.6,0.7) (green line), (*θ*_*d*_,*θ*_*p*_) = (0.3,0.7) (orange line) and (*θ*_*d*_,*θ*_*p*_) = (0.1,0.7) (blue line). (B-D) Pre- and post-spiking is triggered by a single pulse with Δ*t* = −0.02 *sec* in (B), Δ*t* = 0 in (C) and Δ*t* = 0.02 in (D) for (*θ*_*d*_,*θ*_*p*_) = (0.3,0.7). The corresponding behaviour of [*Ca*] and *ρ* are shown in the middle and bottom panels, respectively. The orange dotted and green dotted lines in the middle panels mark *θ*_*d*_ and *θ*_*p*_, respectively.

In our model, long-term plasticity depends on the time lag between the interictal spikes of NMM_1_ and NMM_2_, as well. Here, the term “spike” refers to shape of the model output assumed as the sum of the input signals to the subpopulation P of NMM_i_ (see Section The revisited neural mass model). In this section we assume that only the physiological synaptic plasticity mechanism (Fig 3A) is active. Fig 5 exemplifies how *ρ* changes in response to single spikes of NMM_1_ and NMM_2_ elicited by a pulse stimulation when *ρ* is initiated at *ρ*(0) = 0.5. The synaptic variable *ρ* potentiates regardless of the pulse delay for (*θ*_*d*_,*θ*_*p*_) = (0.6,0.7), depresses (*θ*_*d*_,*θ*_*p*_) = (0.1,0.7) (Fig 5A). We obtain a depression-potentiation-depression curve of Δ*ρ* for (*θ*_*d*_,*θ*_*p*_) = (0.3,0.7), for which the system response is shown in Fig 5B–5D. When NMM_2_ spikes Δ *t* = 0.02 *sec* before NMM_1_, the calcium concentration stays between (*θ*_*d*_,*θ*_*p*_) and *ρ* decreases (Fig 5B). When NMM_2_ and NMM_1_ spike simultaneously, the calcium concentration exceeds *θ*_*p*_ and *ρ* increases (Fig 5C). Finally, when NMM_2_ spikes Δ *t* = 0.02 *sec* after NMM_1_, *ρ* decreases since the calcium concentration stays between (*θ*_*d*_,*θ*_*p*_) (Fig 5D).

**Fig 6:**
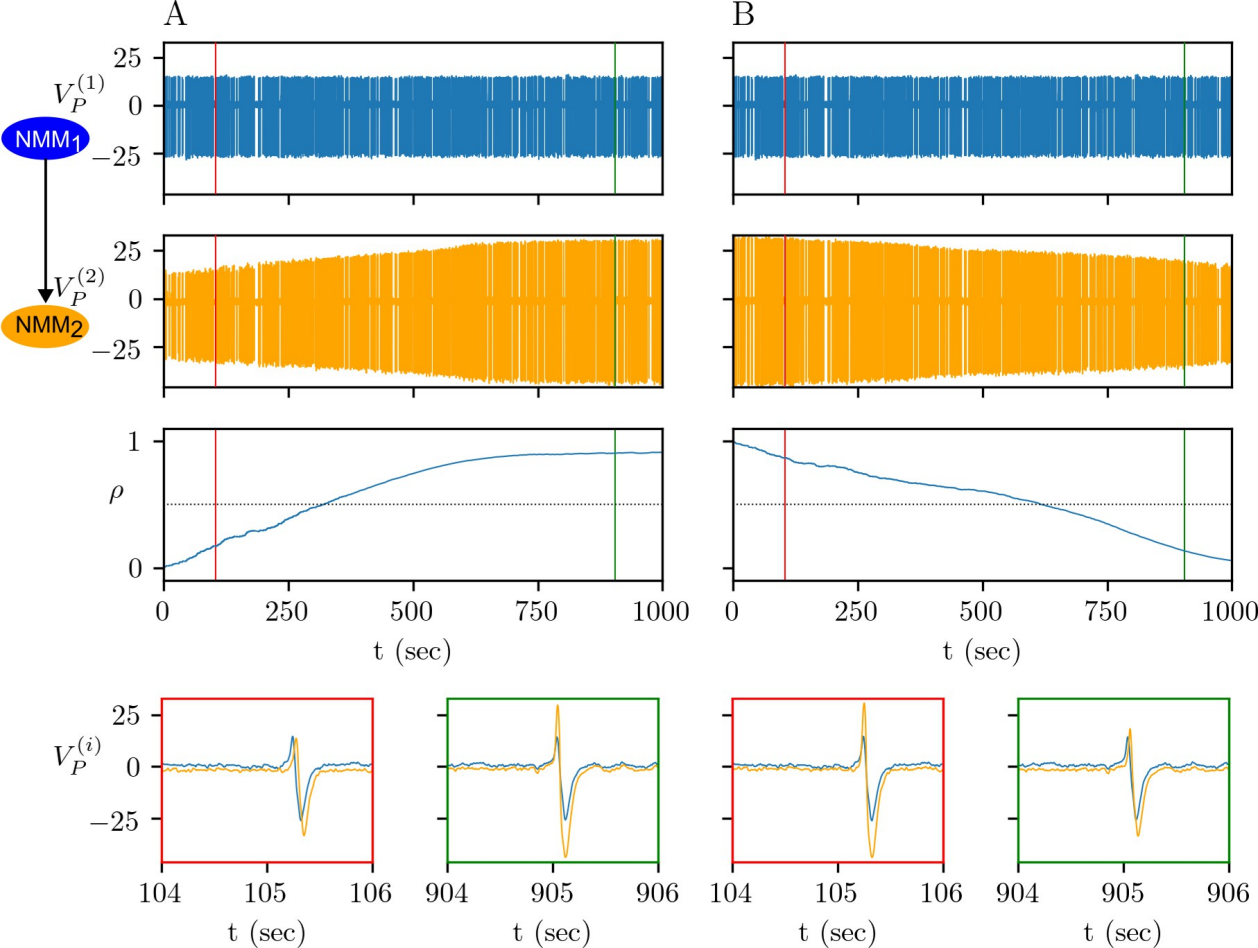
Physiological plasticity under interictal epileptic discharges. (A) Potentiation of initially depressed synapses for (*θ*_*d*_,*θ*_*p*_) = (0.3,0.4). (B) Depression of initially potentiated synapses for (*θ*_*d*_,*θ*_*p*_) = (0.1,1.5).

Depending on the potentiation and depression thresholds, and intrinsic dynamics of the interacting populations, aperiodic but continuous interictal epileptic discharges can cause long-term changes in the presynaptic release probability and coupling strength between the interacting populations (Fig 6). Here, we assume that NMM_1_ is in interictal spiking mode as in Fig 2B, and the less excitable NMM_2_ responds to NMM_1_ by generating spikes. If the potentiation threshold is high, then initially depressed synapses between NMM_1_ and NMM_2_ remain depressed. For a lower potentiation threshold, the depolarization in NMM_2_ is high enough to increase the calcium concentration above *θ*_*p*_, hence causing a positive change in the synaptic variable *ρ*(Δ*ρ*>0). If this regime is maintained long enough and (*θ*_*d*_,*θ*_*p*_) are in a suitable range for LTP, *ρ* can cross the threshold separating depressed to potentiated states, and a pre- and postsynaptic LTP occurs (Fig 6B). Because of the LTP, the time lag between the NMM_1_ and NMM_2_ spikes decreases. In the same configuration but with potentiated synapses, increasing the difference between *θ*_*d*_ and *θ*_*p*_ causes pre- and postsynaptic LTD since the calcium concentration remains in (*θ*_*d*_,*θ*_*p*_) (Fig 6C). Consequently, the time lag between the NMM_1_ and NMM_2_ spikes increases. If both depression and potentiation thresholds are high, despite the interaction between NMM_1_ and NMM_2_, synaptic variables do not change.

**Fig 7:**
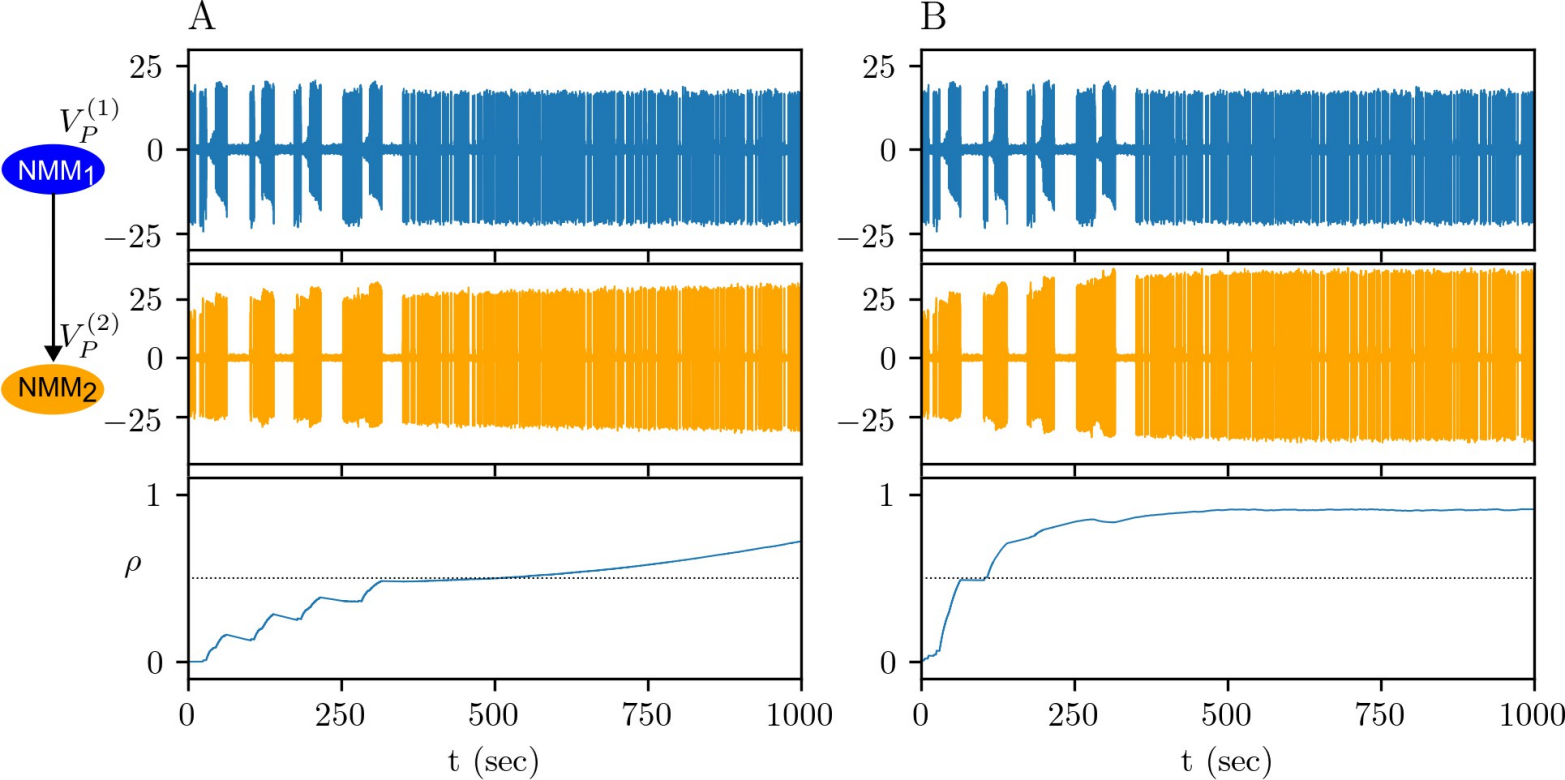
Impact of seizure to physiological plasticity. Potentiation of depressed synapses for (*θ*_*d*_,*θ*_*p*_) = (0.3,0.7) (A) and for (*θ*_*d*_,*θ*_*p*_) = (0.3,0.4) (B).

Experimental studies suggest that a high rate of synchronized activity, during an epileptic seizure for instance, increases the glutamate release. Indeed, the model also suggests that an increased firing rate during a seizure in NMM_1_ can drive the calcium concentration above *θ*_*p*_, and cause LTP, while the interictal activity with the same values of (*θ*_*d*_,*θ*_*p*_) does not change synaptic efficacy (Fig 6A vs Fig 7A). Alternatively, if calcium concentration is already above *θ*_*p*_, then the increased firing rate can accelerate LTP in NMM_2_ (Fig 6B vs Fig 7B). In Fig 7 the repeated seizures in NMM_1_ are triggered by a stochastic input to the *B*^(1)^ variable.

### Expansion of the epileptogenic network

Physiological plasticity alone increases coupling strength by increasing the probability of neurotransmitter release at the presynaptic site and inserting AMPAR at the postsynaptic site. Unless the extrasynaptic NMDAR are activated at the postsynaptic site, the postsynaptic population can preserve its excitation/inhibition balance, hence, does not undergo seizures. However, the extrasynaptic NMDAR activation triggers different processes that disturb the excitation/inhibition balance by reducing the effect of the GABARergic activity [7,52,53] (Fig 3B).

**Fig 8:**
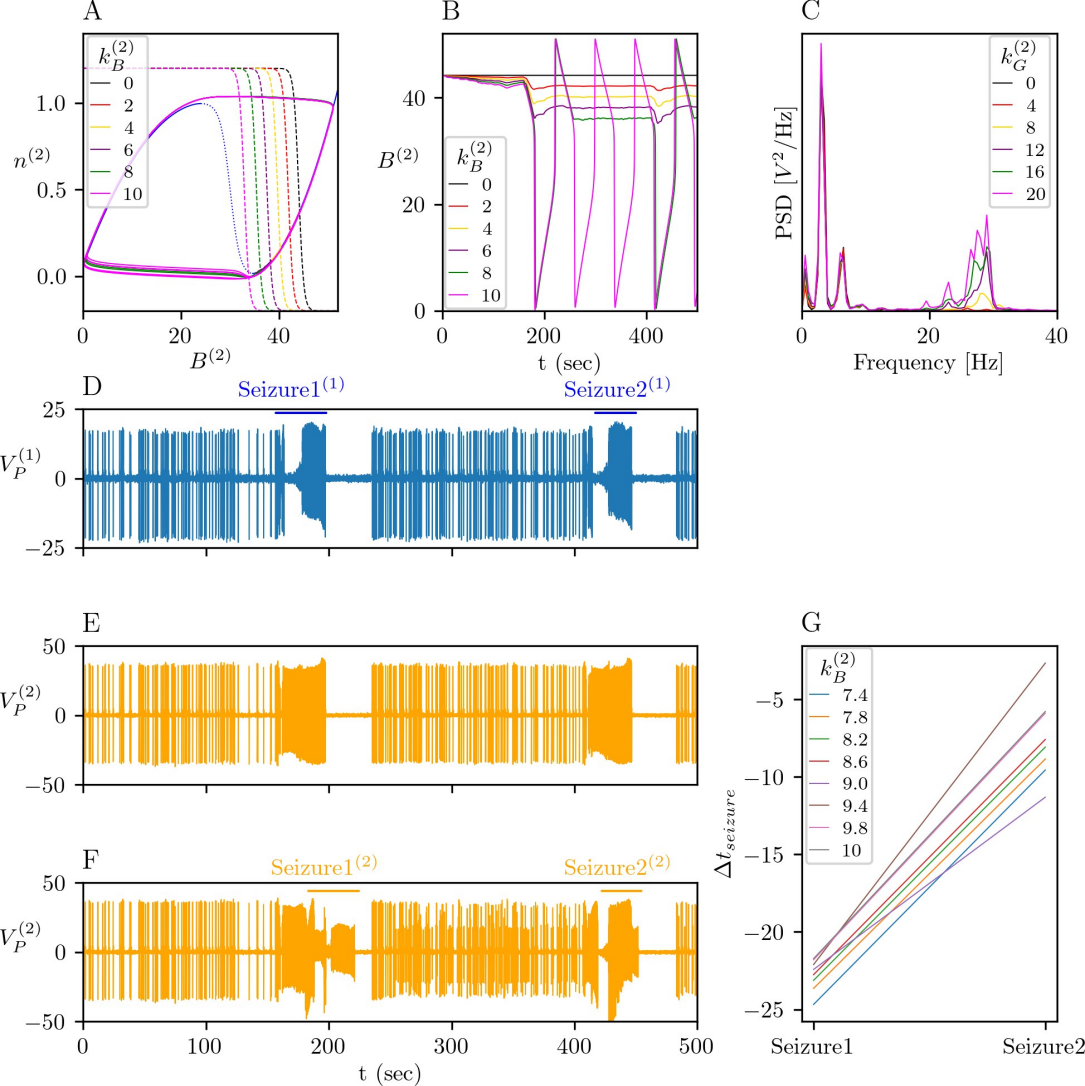
Effect of the pathological plasticity on NMM_2_. (A) Phase portrait of (*B*^(2)^,*n*^(2)^)-subsystem showing the effect of the parameter $k_{B}^{\left(2\right)}$ on the excitability of NMM_2_. NMM_2_ crosses the critical point as $k_{B}^{\left(2\right)}$ increases. (B) Time traces of *B*^(2)^ shown in (A). (C) Normalized PSD of $V_{P}^{\left(2\right)}$ during seizures shows the effect of $k_{G}^{\left(2\right)}$ for $k_{B}^{\left(2\right)}=9$. Gamma-band activity appears for $k_{G}^{\left(2\right)}>8$. (D-E) Example time trace of (9)-(15). (D) Dynamics of NMM_1_ that exhibits two seizures in *t* = [160,197] and in *t* = [408,447]. (E) Dynamics of NMM_2_ for $\left(k_{B}^{\left(2\right)},k_{G}^{\left(2\right)})=(0,0\right)$. (F) Dynamics of NMM_2_ for $\left(k_{B}^{\left(2\right)},k_{G}^{\left(2\right)})=(9,20\right)$, where NMM_2_ exhibits two seizures in *t* = [180,220] and in *t* = [412,415]. (G) Time differences between the seizures exhibited by NMM.

We assumed that the extrasynaptic NMDAR (14) subtype is activated after the synapses are potentiated, i.e. for *U*_*s*_>0.7. The extrasynaptic NMDAR activity changes the amplitude of the PV interneurons and the excitability of NMM_2_ via the auxiliary variable *K*(*t*) (15). We investigate the impact of these changes in the whole model given by (9)-(15). We simulate the whole system (9)-(15) starting from the last point of the solution in Fig 7B for (*θ*_*d*_,*θ*_*p*_) = (0.3,0.4). When NMM_1_ undergoes seizures, and NMM_2_ responds by epileptic spikes since NMM_2_ has a low excitability. Activation of the extrasynaptic NMDAR drives the variable *K*(*t*) to 0 as it causes “structural" changes within NMM_2_. In particular, the amount of the GABAergic loss in NMM_2_, which is represented by the parameter $k_{B}^{\left(2\right)}$, can decrease the excitablity threshold of NMM_2_ and cause seizures in NMM_2_. The relation between the GABAergic loss and seizure in NMM_2_ is represented in the phase space of (*B*^(2)^,*n*^(2)^) in Fig 8A, and the time traces are shown in Fig 8B. For $0\leq k_{B}^{\left(2\right)}<9.5$, the equilibrium point of the (*B*^(2)^,*n*^(2)^)-subsystem remains on the right of the critical point of the seizure transitions (i.e. right fold of the *B*^(2)^-nullcline). For $0\leq k_{B}^{\left(2\right)}<7.4$, NMM_2_ responds to the seizures of NMM_1_ by interictal spikes only. For $7.4\leq k_{B}^{\left(2\right)}<9.5$, the fluctuations in *K*(*t*) due to the activity of NMM_1_ cause *B*^(2)^ to cross the critical point and to jump to the left branch of the *B*^(2)^-nullcline, which correspond to an ictal regime in NMM_2_. After following the left branch of the *B*^(2)^-nullcline, *B*^(2)^ jumps back to the right branch of the *B*^(2)^-nullcline, hence NMM_2_ to the interictal regime. For $9.5\leq k_{B}^{\left(2\right)}$, NMM_2_ generates spontaneous and periodic seizures as the equilibrium point of the (*B*^(2)^,*n*^(2)^)-subsystem passes to the left of the critical point of the seizure transition.

One of the markers of the seizure onset zone in the case of focal epilepsy is the gamma-band activity observed at the seizure onset [45]. Parameter $k_{G}^{\left(2\right)}$ scales the impact of the variable *K*(*t*), hence of the activation of extrasynaptic NMDAR, on the fast GABAergic interneurons. NMM_2_ generates a gamma-band activity at the seizure onset for $k_{G}^{\left(2\right)}>8$ as demonstrated in the power spectral density (PSD) of $V_{P}^{\left(2\right)}$ during a seizure triggered by NMM_1_ for $k_{B}^{\left(2\right)}=9$ (Fig 8C). Fig 8E and 8F show the response of NMM_2_ for $\left(k_{B}^{\left(2\right)},k_{G}^{\left(2\right)})=(0,0\right)$ and $\left(k_{B}^{\left(2\right)},k_{G}^{\left(2\right)})=(9,20\right)$ for the epileptic spikes and seizure of NMM_1_, respectively. NMM_2_ exhibits a seizure in *t* = [180,220] after spiking as a response to the first seizure in NMM_1_ in *t* = [160,197], and a second one in *t* = [412,451] as a response to the second seizure in NMM_1_ in *t* = [408,447]. The time delay between the first seizure in NMM_1_ and NMM_2_’s response to it ($\Delta t_{seizure1}=t_{seizure1}^{\left(1\right)}-t_{seizure1}^{\left(2\right)}$) varies between -25 sec and -20 sec, whereas the delay between the second seizure in NMM_1_ and NMM_2_’s response ($\Delta t_{seizure2}=t_{seizure2}^{\left(1\right)}-t_{seizure2}^{\left(2\right)}$) varies between -16 sec and -1 sec (Fig 8G). This variation in the response times is due to the difference between the pathological plasticity stages: the first seizure occurs in the early stage (before the transition from *K*≈0 to *K*≈1 in (15)), whereas the second one is in the late stage (after the transition from *K*≈0 to *K*≈1 in (15)), highlighting the progressive nature of the condition and its impact on neural dynamics. We also notice that Δ*t*_*seizure*_ decreases with increasing $k_{B}^{\left(2\right)}$ (Fig 8G), hence as NMM_2_ loses its GABAergic integrity.

### Activity in the secondary focus after silencing the epileptic zone

**Fig 9:**
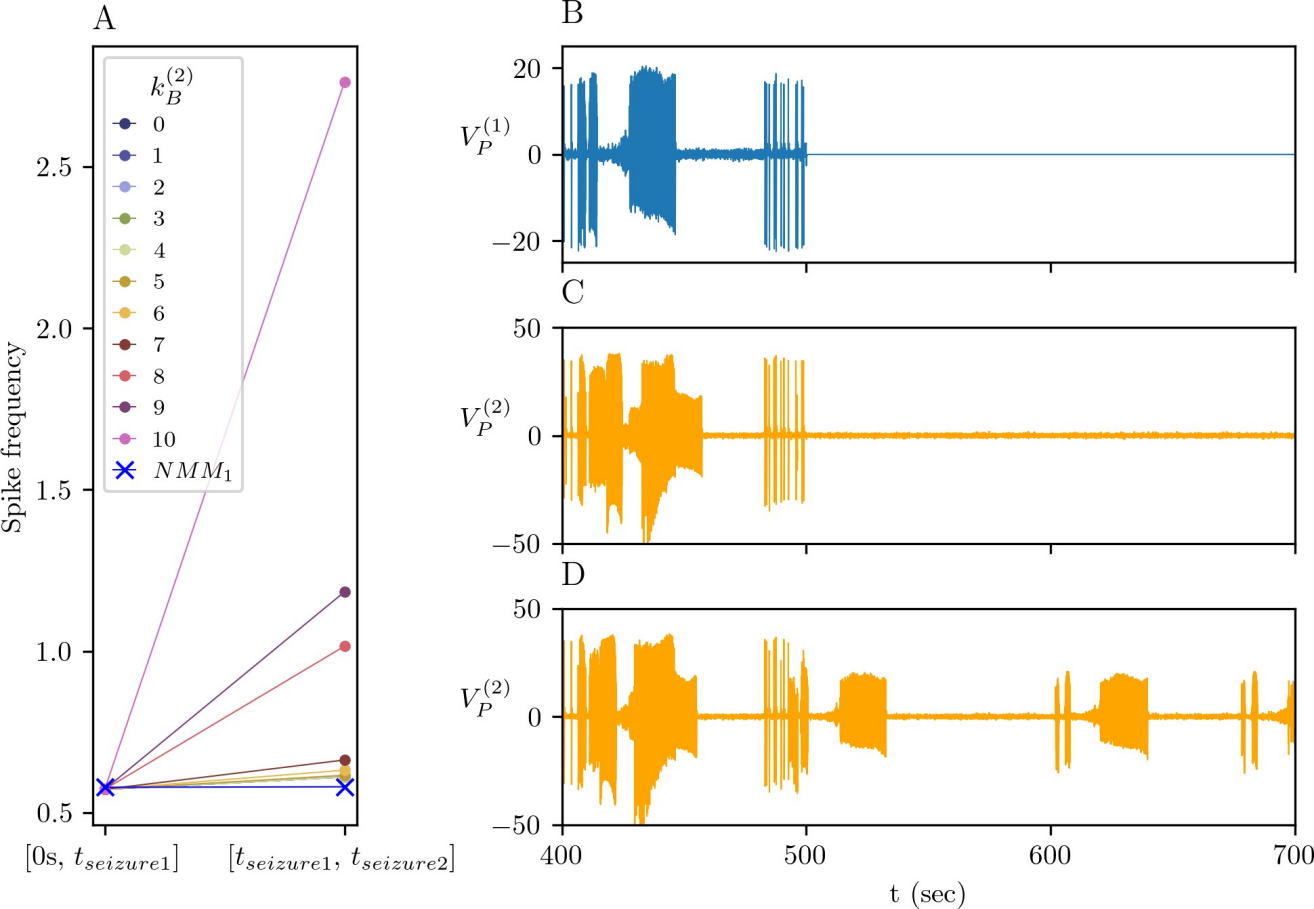
Resection of the primary focus. (A) Spike frequencies of NMM_1_ and NMM_2_ for different values of $k_{B}^{\left(2\right)}$ before NMM_1_’s first seizure between *t* = [0,160] and between NMM_1_’s first and second seizures in *t* = [210,400] in Fig 8. The simulations are continued starting from *t* = 500*sec* of the solutions in Fig 8. After the cessation of any form of activity in NMM_1_ at *t* = 500*sec* (B), NMM_2_ stops exhibiting seizures for $k_{B}^{\left(2\right)}=9$ (C) but continues spontaneous seizures for $k_{B}^{\left(2\right)}=10$ (D).

The activation of extrasynaptic NMDAR induces irreversible structural changes in NMM_2_, in particular, in its GABAergic structure. How significant these changes are can be observed from the difference between the activities of NMM_1_ and NMM_2_. For this we compute the spike frequencies of NMMs before the first seizure in NMM_1_ in *t* = [0,160], which corresponds to the beginning of the pathological plasticity process, and between the first and second seizures in NMM_1_ in *t* = [200,400], which corresponds to the late phase of the pathological plasticity process. The spike frequency of NMM_2_ is computed for the intervals *t* = [0,160] and *t* = [210,400], so after its response to the NMM_1_’s seizures (Fig 9A). The spike frequencies of NMM_1_ in these time windows remain intact. The spike frequencies in NMM_2_ are indistinguishable from the ones of NMM_1_ for $0\leq k_{B}^{\left(2\right)}\leq 7$, but they are much higher for $k_{B}^{\left(2\right)}=\{8,9,10\}$. This is because as $k_{B}^{\left(2\right)}$ increases, the excitability threshold of NMM_2_ decreases and the amplitude of fluctuations in *K*(*t*) increases. These two actors trigger additional spikes (Fig 8E) and seizures in NMM_2_ (Fig 8B for $k_{B}^{\left(2\right)}=10$). Then the question is what happens to NMM_2_ if NMM_1_ is silenced. To answer this question, we reinitiate the system (9)-(15) from the last points of the solutions in Fig 8 for $A^{\left(1\right)}=0$ (Fig 9B–9D). Removing the input from NMM_1_ to NMM_2_ abolishes interictal spikes in NMM_2_ both for $k_{B}^{\left(2\right)}=9$ and $k_{B}^{\left(2\right)}=10$ and seizures for $k_{B}^{\left(2\right)}=9$. If the GABAergic loss is important, as it is for $k_{B}^{\left(2\right)}=10$, stochastic inputs to *B*^(2)^(*t*) can trigger spontaneous seizures NMM_2,_ despite a completely silent NMM_1_.

## Discussion

In this study, we presented a NMM of physiological and pathological plasticity under epileptic activity. Physiological plasticity takes into account short- and long-term variations in the glutamatergic interactions at both presynaptic and postsynaptic sites. Pathological plasticity considers the mechanisms that can contribute to abnormal changes resulting in exaggerated hyperexcitability associated with epileptic activity, such as GABAergic modulations at the postsynaptic site. Under epileptic activity, increased connectivity strength resulting from the physiological plasticity triggers the pathological plasticity, which can cause a secondary focus. These mechanisms have been implemented in an autonomous (self-driven) NMM, which generates the typical focal seizure pattern from interictal to seizure termination.

In neurobiological studies, spillover has been observed during synchronous activation of a large number of glutamatergic fibers [54]. NMMs, by definition, represent synchronous activity of a neural population and therefore synchronicity is implicitly coded in the model [55]. Physiological synaptic plasticity in the model integrates three main mechanisms. The first one is the presynaptic STP. The second one is the calcium-mediated long-term synaptic plasticity that changes the probability of glutamate release at the presynaptic site and insertion of AMPAR at the postsynaptic site. The third mechanism is the consolidation of the long-term plasticity. Although the description of consolidation remained phenomenological as in [30,32], its integration into the model allowed for a sustained change in synaptic connectivity.

We assumed that presynaptic and postsynaptic long-term plasticity are proportional. While this simplified assumption is true for retrograde nitric oxide signaling, retrograde endocannabinoid release works in the opposite direction. Costa et al. [37] suggested a phenomenological model integrating the effects of opposing proteins on long-term presynaptic plasticity. Such effects can be integrated in our model, for instance, by introducing a second variable to track the calcium variations, which modifies the neurotransmitter release probability.

Our approach of modeling NMDA currents is quasi-physiological, similar to [38], but without explicitly modeling the magnesium gate. Yet, it does include the essential ingredients mediating the NMDAR activity, which are the presynaptic release and postsynaptic potentiation. Conductance-based modeling of synaptic receptors in the neural mass formulation has been proposed in [56], and more recently, in [57]. While the conductance-based neural mass formulation may provide a more physiological way of modeling neural dynamics, we argue that our approach is consistent with the neural mass formulation we considered and captures the key ingredients necessary for the problem studied. Interictal epileptic discharges in the presynaptic population and the postsynaptic response to them can induce LTP or LTD depending on the plasticity thresholds. The synchronization between pre- and postsynaptic interictal epileptic discharges increases (decreases) with LTP (LTD). Ictal activity in the presynaptic population speeds up the LTP process, and if frequent, it can cause a transition from LTD-trend to LTP-trend. LTP then triggers pathological mechanisms as the postsynaptic region receives higher glutamatergic input from the presynaptic region. The model suggests that preventing LTP by targeting both the presynaptic region (decreasing the epileptic activity) and postsynaptic region (blocking NMRA receptors, increasing the potentiation threshold, or applying GABAergic agonists, etc.) can be crucial to avoid pathological consequences.

The pathological plasticity under epileptic activity includes disruption of the GABAergic pathway caused by the activation of the extrasynaptic NMDAR. The model captures possible changes without detailing the process, such as how calcium entry through the extrasynaptic NMDAR acts on calpain, how a decreasing number of KCC2 changes the GABA reversal potential, or how GABAR expression is downregulated, etc. Rather, the impact of the extrasynaptic NMDAR activation is reflected by the auxiliary variable *K*(*t*) that acts on the perisomatic and dendritic GABAergic interneurons of the NMM. The former mimics the effect of KCC2 downregulation at the level of interactions between PV interneurons and from PV interneurons to PYR cells that induces a gamma-band activity, which is considered a signature of an epileptogenic zone in focal seizures [58–60]. The latter mimics the effect of GABAR downregulation that reduces the excitability threshold of the population targeted by the epileptic population. Indeed, the dendritic SOM interneurons control the calcium dynamics, for instance by directly inhibiting calcium influx [61–63]. Low dendritic SOM projection may lead to accumulation of intracellular calcium and aggravate KCC2 dysfunctions. The impact of the extrasynaptic NMDAR activation on the GABAergic system is scaled in the model. This scaling factor can be interpreted as an intrinsic property of the region subject to epileptic activity that can maintain the GABAergic integrity. In our simulations, we observe a delay between the seizures when a seizure in the postsynaptic region is triggered by a presynaptic seizure. Such propagation delays have been reported in animal models of focal epilepsy [64], and interestingly, it can decrease during epileptogenesis [65].

In this study we extended a widely used model of the hippocampus [40] for ensuring autonomous epileptic activity from interictal to ictal and seizure termination. Essentially, this was achieved by introducing a slow subsystem that modulates the IPSP amplitude of the slow GABAergic interneurons. In other words, we introduced a slow-fast process for obtaining an autonomous interictal-to-ictal transition. Except for very few studies [59,66], the parameter variations leading to an interictal-to-ictal transition has been performed manually, in particular by varying the IPSP amplitude of the slow GABAergic interneurons. In [58,59] a dynamic chloride accumulation in pyramidal neurons caused by GABAergic activity modulates the IPSP amplitude. However, the authors of [59] do not propose a mechanism for seizure termination. In [66] this modulation was obtained through feedback from the activity of the pyramidal cells. Our approach is similar to the phenomenological approach of [66], except the feedback from the pyramidal neurons and the dimension of the slow subsystem. In the transition to seizures, not only pyramidal neurons but also interneurons can play a critical role [67]. Our model can be improved further to reflect the involvement of specific cellular and network mechanisms by considering appropriate feedback mechanisms. Furthermore, the fast subsystem representing neuronal subpopulations and the slow subsystem controlling the excitability and the IPSP amplitude have the essential ingredients for studying different bifurcation types, which can be inherent to specific seizure types (absence seizures, focal seizures) with different seizure onset patterns [45]. Furthermore, the slow-fast system can be used to study the effect of electrical stimulation that is a general procedure in clinics to understand the excitability of the brain region to identify epileptogenic networks.

Our study of secondary epileptogenesis confirms the so-called *seizures can beget seizures* phenomena: epileptic activity in one brain region can recruit healthy regions and cause the formation of a complex epileptic network [68]. The model suggests that epileptic seizures boosts physio-pathological plasticity, as suggested in the literature [8,69]. Once a healthy zone is driven to the critical point, then it generates epileptic discharges and/or seizures. The phenomenon of secondary epileptogenesis was first demonstrated in animal models [49], including: (1) the electrical stimulation kindling-like model in frogs, rabbits, rats, guinea pigs, and cats; (2) the kainic acid model in rats and mice; and (3) several others [27]. In humans, this phenomenon can be observed in clinical practice [27], and it can be related to the organization of epileptic networks. The network concept in epilepsy can be a key factor in identifying the anatomic distribution of the epileptogenic process and in the clinical expression of dynamic course of seizures [70,71]. For example, development of the epileptic networks in temporal lobe epilepsy is correlated with time (epilepsy duration) and has been argued to be due to secondary epileptogenesis processes [72,73]. Therefore, the timing of interference with epileptic activity may be critical [74]. Indeed, in clinical practice, acute symptomatic seizures are treated with anti-seizure medication [75]. Furthermore, the secondary epileptogenesis is not only limited to the “creation” of a secondary focus or the expansion of an epileptic network but also to the expansion of the dysfunctional tissues. For instance, the epileptic zone can be larger that MRI-visible lesion, which can require larger resections than the lesion [76,77], and related the abnormalities in surrounding cortex [78]. In addition to the role of epileptic activity in epileptogenesis, epileptic activity can interfere with physiological oscillatory activity in distributed neural networks and cause cognitive comorbidity [79–83]. Generalizing our model to personalized dynamic brain network modeling for planning the treatment of epilepsy is a future research direction [84].

On the other hand, epilepsy is a complex and patient-specific disease, and it is challenging to propose a “one-fits-all” theory. The “seizures beget seizures” phenomenon has been questioned recently [85,86]. The correlation between epilepsy duration and number of high epileptogenic structures was not reported for every case. This suggests that mechanisms of epileptic network’s extension (and most likely of secondary epileptogenesis) rely on many factors, such as the intrinsic properties of underlying lesion and the anatomical substrate of the location of the primary epileptogenic zone. Our model has many limitations to address this challenge. First, it only considers glutamatergic plasticity mediated by epileptic activity between unidirectionally coupled neuronal populations. A growing body of literature suggests that inhibitory GABAergic synapses exhibit long-term plasticity, which can have a protective role against the expansion of epileptogenic networks [87,88]. Local GABAergic mechanisms, such as blanket inhibition [89,90] and local circuity [91], can serve to balance excitation and prevent epilepsy. The model does not address mGLUR mediated plasticity [92,93], homeostatic effects [94,95] or other mechanisms, such as epigenetic changes [96]. Another limiting factor can be the structural and pathological differences between the primarily and secondary regions. Indeed, the impact of the pathological changes are scaled in the model, which can be considered to study the propagation of the epileptic activity in a network of connected nodes with different levels of GABAergic integrity. Yet here, we restricted ourselves to a minimal network of glutamatergic feedforward connectivity, where the projection is unidirectional on excitatory neuronal subpopulation. The glutamatergic interactions between brain regions are often bidirectional targeting both excitatory neurons and inhibitory interneurons. Inhibitory populations can also have long-range inhibitory projections [97,98]. These inhibitory/excitatory bidirectional interactions would eventually impact the network dynamics under epileptic activity. In addition, the considered NMM is the simplest formulation for the CA1 region of the hippocampus. A laminar NMM can be considered for the distant synaptic rules related to dendritic location, which can be useful for studying the plasticity induced in neocortical regions by non-invasive brain stimulation. Modeling experimental recording could be an asset. Finally, the model simulates epileptiform discharges, whereas the physiological plasticity is also mediated by theta rhythm and theta-gamma oscillations in the hippocampus [99]. These points will be addressed in future works.

## Conclusion

We have proposed a conceptual framework based on neural mass modeling for studying physio-pathological plasticity under epileptic activity. We have shown how strengthened connectivity between an epileptic and non-epileptic brain regions can lead to lead to GABAergic dysfunctions in the latter, and it can form an epileptogenic network. Our study can benefit the development of *plastic* large-scale brain models for studying the expansion of epileptogenic networks, formation of secondary foci, and the design of invasive or non-invasive brain stimulation protocols to control the epileptogenic networks.

## Supporting information

S1 FigDynamics of NMM_1_ and NMM_2_ during the interictal phase under a unidirectional interaction from NMM_1_ to NMM_2_ for the parameter set given in Table 2 of the main text.(A) Bifurcation diagram of NMM_1_ where the amplitude of $y_{P}^{\left(1\right)}$ is presented as a function of *B*^(1)^. The blue curve shows the branch of equilibrium points (bold for stable and dashed for unstable equilibrium points). The red curves show the amplitude of $y_{P}^{\left(1\right)}$ in the oscillatory regime. The Hopf bifurcations along the branch of equilibrium points are denoted by red dots and saddle-node bifurcation by blue dots. The dynamical regimes that correspond to the fast onset, ictal and interictal periods are marked by purple, yellow and cyan patches, respectively. The time solution (black curve) is superimposed on the bifurcation diagram. (B) Time trace for $V_{P}^{\left(1\right)}$ showing interictal spikes. (C) Phase plane of the (*B*^(1)^,*n*^(1)^)-subsystem with the *B*^(1)^-nullcline (blue curve, bold for stable and dashed for branches) and the *n*^(1)^-nullcline (orange curve). The *B*^(1)^ values that correspond to the dynamical regimes in (A) are marked by the same color code. The time solution (black curve) is superimposed on the phase plane. (E) Bifurcation diagram of the uncoupled NMM_2_ where the amplitude of $y_{P}^{\left(2\right)}$ is presented as a function of *B*^(2)^. The blue curve shows the branch of equilibrium points (bold for stable and dashed for unstable equilibrium points). The red curves show the amplitude of $y_{P}^{\left(2\right)}$ in the oscillatory regime. The Hopf bifurcations along the branch of equilibrium points are denoted by red dots and saddle-node bifurcation by blue dots. The dynamical regimes that correspond to the oscillatory states is marked in yellow, the steady states in orange for low values of *B*^(2)^ and in cyan for high values of *B*^(2)^. The time solution (black curve) is superimposed on the bifurcation diagram. (F) Time trace for $V_{P}^{\left(2\right)}$ showing interictal spikes. (G) Phase plane of the (*B*^(2)^,*n*^(2)^)-subsystem with the *B*^(2)^-nullcline (blue curve, bold for stable and dashed for branches) and the *n*^(1)^-nullcline (orange curve). The *B*^(2)^ values that correspond to the dynamical regimes in (E) are marked by the same color code. The time solution (black curve) is superimposed on the phase phane.(TIF)

S2 FigDynamics of NMM_1_ and NMM_2_ during the ictal phase under a unidirectional interaction from NMM_1_ to NMM_2_ for the parameter set given in Table 2 of the main text.(A) Bifurcation diagram of NMM_1_ where the amplitude of $y_{P}^{\left(1\right)}$ is presented as a function of *B*^(1)^. The blue curve shows the branch of equilibrium points (bold for stable and dashed for unstable equilibrium points). The red curves show the amplitude of $y_{P}^{\left(1\right)}$ in the oscillatory regime. The Hopf bifurcations along the branch of equilibrium points are denoted by red dots and saddle-node bifurcation by blue dots. The dynamical regimes that correspond to the fast onset, ictal and interictal periods are marked by purple, yellow and cyan patches, respectively. The time solution (black curve) is superimposed on the bifurcation diagram. (B) Time trace for $V_{P}^{\left(1\right)}$ showing interictal spikes. (C) Phase plane of the (*B*^(1)^,*n*^(1)^)-subsystem with the *B*^(1)^-nullcline (blue curve, bold for stable and dashed for branches) and the *n*^(1)^-nullcline (orange curve). The *B*^(1)^ values that correspond to the dynamical regimes in (A) are marked by the same color code. The time solution (black curve) is superimposed on the bifurcation diagram. (E) Bifurcation diagram of the uncoupled NMM_2_ where the amplitude of $y_{P}^{\left(2\right)}$ is presented as a function of *B*^(2)^. The blue curve shows the branch of equilibrium points (bold for stable and dashed for unstable equilibrium points). The red curves show the amplitude of $y_{P}^{\left(2\right)}$ in the oscillatory regime. The Hopf bifurcations along the branch of equilibrium points are denoted by red dots and saddle-node bifurcation by blue dots. The dynamical regimes that correspond to the oscillatory states is marked in yellow, the steady states in orange for low values of *B*^(2)^ and in cyan for high values of *B*^(2)^. The time solution (black curve) is superimposed on the phase plane. (F) Time trace for $V_{P}^{\left(2\right)}$ showing interictal spikes. (G) Phase plane of the (*B*^(2)^,*n*^(2)^)-subsystem with the *B*^(2)^-nullcline (blue curve, bold for stable and dashed for branches) and the *n*^(1)^-nullcline (orange curve). The *B*^(2)^ values that correspond to the dynamical regimes in (A) are marked by the same color code. The time solution (black curve) is superimposed on the phase plane.(TIF)
