# Supplementary figures and images for: Expansion of epileptogenic networks via neuroplasticity in neural mass models
Source: PLoS Comput Biol. 2024 Dec 3;20(12):e1012666. doi: 10.1371/journal.pcbi.1012666 (PMC11642990; doi:10.1371/journal.pcbi.1012666)

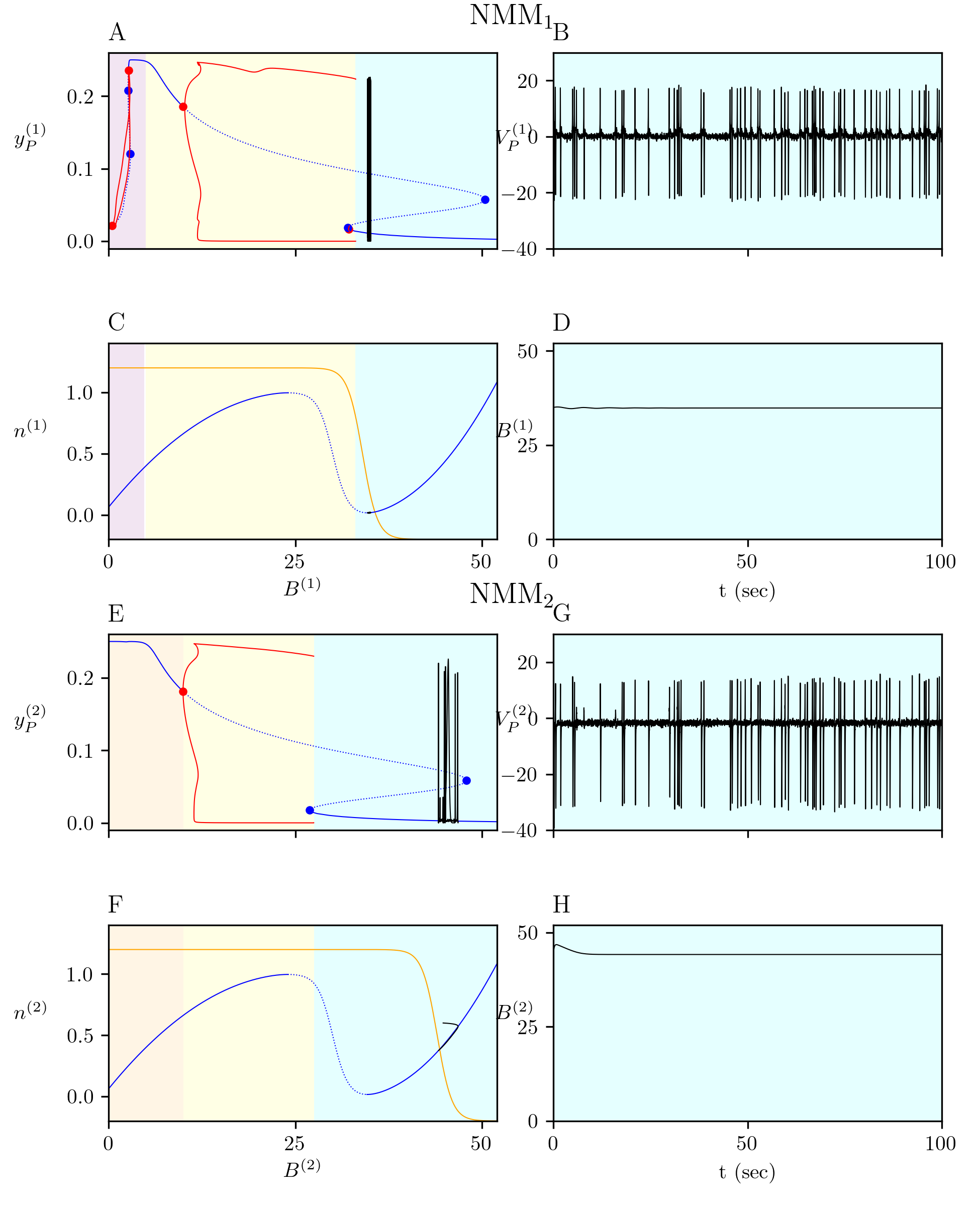

Supplement: S1 Fig — (A) Bifurcation diagram of NMM1 where the amplitude of yP(1) is presented as a function of B(1). The blue curve shows the branch of equilibrium points (bold for stable and dashed for unstable equilibrium points). The red curves show the amplitude of yP(1) in the oscillatory regime. The Hopf bifurcations along the branch of equilibrium points are denoted by red dots and saddle-node bifurcation by blue dots. The dynamical regimes that correspond to the fast onset, ictal and interictal periods are marked by purple, yellow and cyan patches, respectively. The time solution (black curve) is superimposed on the bifurcation diagram. (B) Time trace for VP(1) showing interictal spikes. (C) Phase plane of the (B(1),n(1))-subsystem with the B(1)-nullcline (blue curve, bold for stable and dashed for branches) and the n(1)-nullcline (orange curve). The B(1) values that correspond to the dynamical regimes in (A) are marked by the same color code. The time solution (black curve) is superimposed on the phase plane. (E) Bifurcation diagram of the uncoupled NMM2 where the amplitude of yP(2) is presented as a function of B(2). The blue curve shows the branch of equilibrium points (bold for stable and dashed for unstable equilibrium points). The red curves show the amplitude of yP(2) in the oscillatory regime. The Hopf bifurcations along the branch of equilibrium points are denoted by red dots and saddle-node bifurcation by blue dots. The dynamical regimes that correspond to the oscillatory states is marked in yellow, the steady states in orange for low values of B(2) and in cyan for high values of B(2). The time solution (black curve) is superimposed on the bifurcation diagram. (F) Time trace for VP(2) showing interictal spikes. (G) Phase plane of the (B(2),n(2))-subsystem with the B(2)-nullcline (blue curve, bold for stable and dashed for branches) and the n(1)-nullcline (orange curve). The B(2) values that correspond to the dynamical regimes in (E) are marked by the same color code. [file pcbi.1012666.s001.tif]

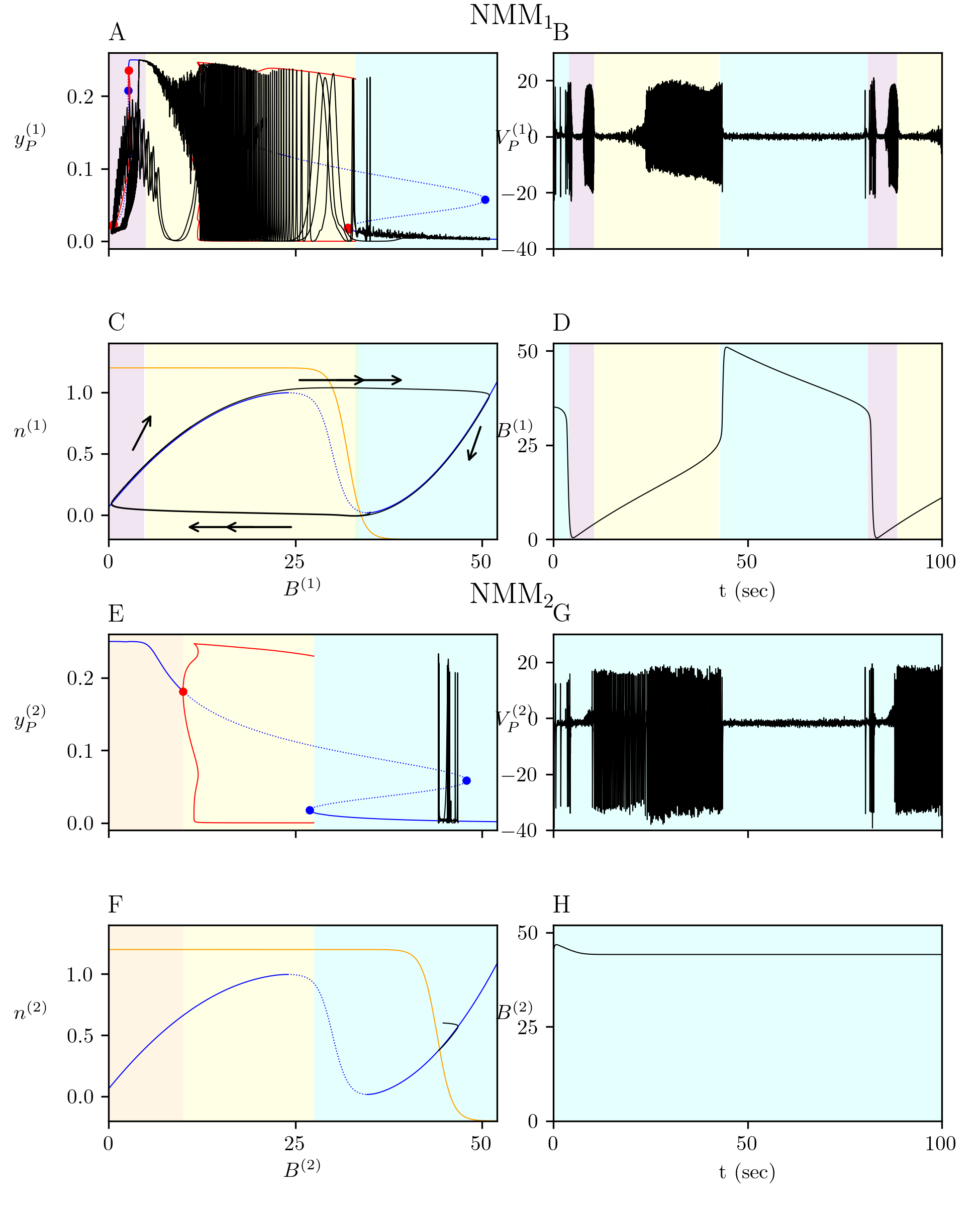

Supplement: S2 Fig — (A) Bifurcation diagram of NMM1 where the amplitude of yP(1) is presented as a function of B(1). The blue curve shows the branch of equilibrium points (bold for stable and dashed for unstable equilibrium points). The red curves show the amplitude of yP(1) in the oscillatory regime. The Hopf bifurcations along the branch of equilibrium points are denoted by red dots and saddle-node bifurcation by blue dots. The dynamical regimes that correspond to the fast onset, ictal and interictal periods are marked by purple, yellow and cyan patches, respectively. The time solution (black curve) is superimposed on the bifurcation diagram. (B) Time trace for VP(1) showing interictal spikes. (C) Phase plane of the (B(1),n(1))-subsystem with the B(1)-nullcline (blue curve, bold for stable and dashed for branches) and the n(1)-nullcline (orange curve). The B(1) values that correspond to the dynamical regimes in (A) are marked by the same color code. The time solution (black curve) is superimposed on the bifurcation diagram. (E) Bifurcation diagram of the uncoupled NMM2 where the amplitude of yP(2) is presented as a function of B(2). The blue curve shows the branch of equilibrium points (bold for stable and dashed for unstable equilibrium points). The red curves show the amplitude of yP(2) in the oscillatory regime. The Hopf bifurcations along the branch of equilibrium points are denoted by red dots and saddle-node bifurcation by blue dots. The dynamical regimes that correspond to the oscillatory states is marked in yellow, the steady states in orange for low values of B(2) and in cyan for high values of B(2). The time solution (black curve) is superimposed on the phase plane. (F) Time trace for VP(2) showing interictal spikes. (G) Phase plane of the (B(2),n(2))-subsystem with the B(2)-nullcline (blue curve, bold for stable and dashed for branches) and the n(1)-nullcline (orange curve). The B(2) values that correspond to the dynamical regimes in (A) are marked by the same color code. [file pcbi.1012666.s002.tif]
